# Operant conditioning deficits and modified local field potential activities in parvalbumin-deficient mice

**DOI:** 10.1038/s41598-021-82519-3

**Published:** 2021-02-03

**Authors:** Alessandra Lintas, Raudel Sánchez-Campusano, Alessandro E. P. Villa, Agnès Gruart, José M. Delgado-García

**Affiliations:** 1 Neuroheuristic Research Group & LABEX, HEC Lausanne, University of Lausanne, Quartier UNIL-Chamberonne, 1015 Lausanne, Switzerland; 2 Division of Neurosciences, Pablo de Olavide University, Ctra. de Utrera, km. 1, 41013 Sevilla, Spain

**Keywords:** Learning and memory, Operant learning

## Abstract

Altered functioning of GABAergic interneurons expressing parvalbumin (PV) in the basal ganglia-thalamo-cortical circuit are likely to be involved in several human psychiatric disorders characterized by deficits in attention and sensory gating with dysfunctional decision-making behavior. However, the contribution of these interneurons in the ability to acquire demanding learning tasks remains unclear. Here, we combine an operant conditioning task with local field potentials simultaneously recorded in several nuclei involved in reward circuits of wild-type (WT) and PV-deficient (PVKO) mice, which are characterized by changes in firing activity of PV-expressing interneurons. In comparison with WT mice, PVKO animals presented significant deficits in the acquisition of the selected learning task. Recordings from prefrontal cortex, nucleus accumbens (NAc) and hippocampus showed significant decreases of the spectral power in beta and gamma bands in PVKO compared with WT mice particularly during the performance of the operant conditioning task. From the first to the last session, at all frequency bands the spectral power in NAc tended to increase in WT and to decrease in PVKO. Results indicate that PV deficiency impairs signaling necessary for instrumental learning and the recognition of natural rewards.

## Introduction

Parvalbumin (PV) is a cytosolic $\hbox {Ca}^{2+}$-binding protein expressed in a subset of neurons accounting for almost half of all GABAergic interneurons in the brain^1^. PV is also highly expressed in fast-twitch muscles, where it plays a key role in the muscle relaxation rate^2^. The integrity of $\hbox {Ca}^{2+}$-signaling in PV-expressing interneurons is necessary for a correct regulation of neural signaling^3^. Knockout mice for PV (PVKO) have been used for more than two decades as a study model to investigate the effects of the disruption of the proper functioning of PV-expressing interneurons in neural networks^1^. PV-expressing interneurons are characterized by fast-firing properties affecting synchronization of electrical activity in cortical networks^4^ and short-term plasticity in the cerebellum and striatum^5,6^. Moreover, PV-downregulation is sufficient to induce crucial changes in precision of spike timing leading to changes in firing pattern in the thalamo-cortical network^7,8^. Synchronous recruitment of fast-spiking PV-expressing interneurons generated $\gamma$-oscillations during performance of cognitive tasks, thus supporting the key role played by these neurons in $\gamma$-rhythm generation^9^.

The lack of PV in knockout mice yields a phenotype of locomotor activity and coordination characterized by a decreased exploratory activity and by an increased tendency to move in one direction for larger distances and longer intervals before stopping or turning^10^. PV-deficient mice have been shown to develop highly coherent oscillatory activity that can develop into epileptic seizures with high incidence of mortality^4^. Rodent models of schizophrenia are characterized by dysregulation of GABAergic interneurons^11^ and by deficits in sensory-evoked $\gamma$-oscillations^12^. In human schizophrenic patients, it is well established that illness symptoms correlate positively with alterations of $\gamma$-band activity in several experimental paradigms^13,14^. In addition to schizophrenia, the key role of PV-expressing interneurons^15^ has been associated with other mouse models of neurodevelopmental disorders, i.e. Autism Spectrum Disorders (ASD)^16^, Rett syndrome^17^, and Huntington Disease^18^. What is common across these neuropsychological disorders is that they are all characterized by deficits in attention and sensory gating, thus leading to learning and memory deficits. These findings suggest that PV-deficient mice offer as a suitable model for studying the role of PV-expressing interneurons in regional oscillatory activity and in fundamental sensorimotor and cognitive processes expressed during the acquisition of complex learning tasks. A typical experimental paradigm for this kind of study is the operant conditioning^19^.

In the present study, we have checked the behavioral abilities of PVKO and WT mice to solve an operant conditioning using a light/dark paradigm described elsewhere^20^. As already reported, this experimental task involved the participation of different cortical and subcortical structures including the medial prefrontal cortex (mPFC), the CA1 area of the dorsal hippocampus and the nucleus accumbens (NAc), and related nuclei^21^. The medial septum (MS) is a major source of cholinergic projections to the hippocampus critical for the acquisition of Pavlovian and operant conditioning paradigms^22,23^. The output nuclei of the basal ganglia send projections to a number of thalamic nuclei, including the mediodorsal (MD) nucleus of the thalamus, whose lesion provokes impairment in operant conditioning^24,25^. All these structures are rich in PV-expressing interneurons^26–28^. In order to determine ongoing changes in the oscillatory activity across the learning process, we recorded simulteneous local field potential (LFP) profiles from electrodes implanted in the mPFC, the NAc, the hippocampal CA1 area, the MS and the thalamic MD nucleus. When compared with WT mice, PVKO animals presented a substantial deficit in the acquisition of the selected operant conditioning task and important differences in the profiles and spectral powers of LFPs collected from the mentioned structures.

## Results

### PVKO mice presented a lower performance in the acquisition of an operant conditioning task than their WT littermates

**Figure 1:**
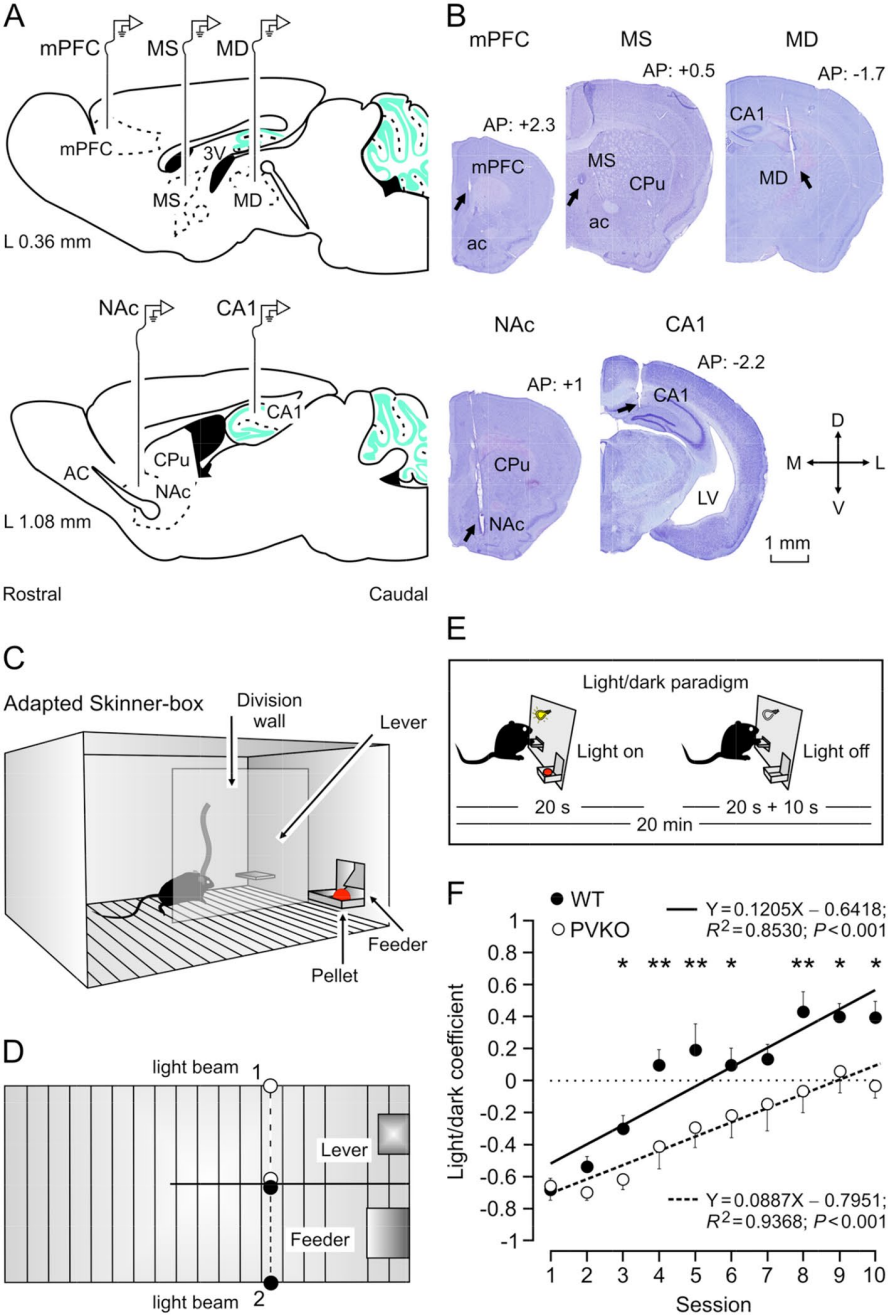
Experimental design and mice performance during the light/dark conditioning paradigm. (**A**) Mice were chronically implanted with single recording electrodes at medial prefrontal cortex (mPFC), medial septum (MS), mediodorsal thalamic nucleus (MD), nucleus accumbens (NAc), and the CA1 area of the dorsal hippocampus (CA1). (**B**) Representative photomicrographs of electrodes implanted in the five recording sites (arrows). Scale bar, 1 mm. ac, anterior commissure; *LV* lateral ventricle; *CPu* caudate-putamen; *AP* anterioposterior; *D* dorsal; *L* lateral; *M* medial; *V* ventral. (**C**) Mice were trained in a modified Skinner box with a Perspex division wall separating the lever from the feeder. (**D**) Each corridor of the modified Skinner box was provided with a light beam located $\approx$ 6 cm from the lever (1) or the feeder (2). At first, all mice (n = 6 WT and n = 6 PVKO) had to acquire a fixed-ratio (1:1) schedule until reaching the criterion set to press the lever for at least 20 times during a 20 min session for 2 successive days. (**E**) After reaching the criterion, the mice were transferred to a light/dark paradigm in which lever presses were reinforced only when a light bulb was switched on. Lever presses performed during the dark period delayed the lighted period for up to an additional 10 s. (**F**) Performance of WT (black dots) and PVKO (white dots) mice (n = 6 per group) during the light/dark test. The light/dark coefficient was calculated according to Eq. (1) (see “Materials and methods”). Values are $mean \pm SEM$ (*$P < 0.05$; **$P < 0.01$). Best linear adjustments to both set of data are illustrated (for more details see Supplementary Table S1).

Following procedures described elsewhere^20,21^, mice (n = 6 mice per group) were initially trained for the acquisition of an operant conditioning task with a fixed-ratio (1:1) schedule—namely, each lever press was reinforced with a food pellet. Training sessions were performed daily and lasted for 20 min (Fig. 1C,D). As indicated in “Materials and methods”, the selected criterion was to press the lever at least 20 times per session for two successive sessions. Animals progressively improved their performance in the Skinner box with the successive sessions and reached the criterion with no differences between groups [WT: $4.6 \pm 0.55$ days; range 3–5 days; PVKO: $3.2 \pm 0.45$ days; range: 3–6 days; Mann-Whitney U statistic; T = 47.000; n(small) = 6; n(big) = 6; *P*(est.) = 0.206; *P* = 0.24]. After reaching the criterion, each animal was trained in a more complex task. In this case, animals were rewarded in a 1:1 fixed-ratio schedule only during the period in which a small light bulb, located over the lever, was switched on (Fig. 1E). Lighted periods lasted for 20 s and were followed by light-off periods during which the animal was not rewarded. Moreover, pressing the lever during the dark period triggered a delay of up to an additional 10 s in the reappearance of the lighted period. The training session lasted for 20 min and was repeated for 10 successive days. As illustrated in Fig. 1F, WT mice acquired this task steadily and progressively across the seven training sessions reaching significantly larger light/dark coefficients than PVKO ones (Two Way Repeated Measures ANOVA *F*-test: $F_{(1,8)}$ = 11.216; $P< 0.01$, followed by the Holm-Sidak test for the multiple comparison procedure, $P \le 0.05$; Fig. 1F). To better illustrate the different performances of the two groups of animals, a regression analysis to the two set of data was applied. A linear model was the best adjustment to the collected data (WT: $\hbox {R}^{2}$ = 0.8530; $P < 0.001$; and PVKO: $\hbox {R}^{2}$ = 0.9368; $P < 0.001$; for more details see Supplementary Table S1).

### A comparison of LFPs recorded in five selected brain sites from PVKO and WT mice during the performance of the selected instrumental task

**Figure 2:**
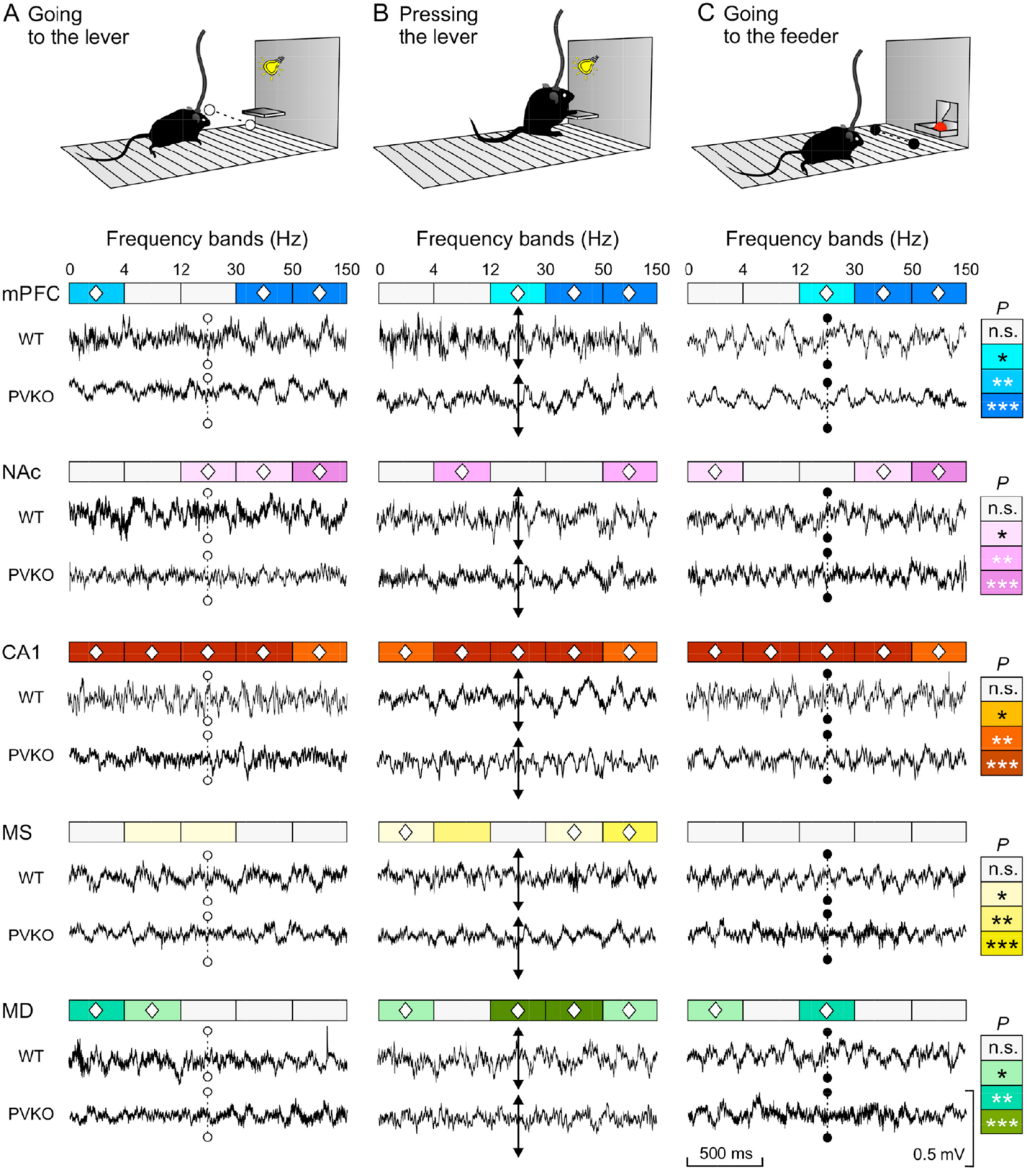
Differences in LFPs recorded at the five selected brain areas in WT and PVKO mice during the performance of the light/dark operant conditioning test. (**A**) Representative LFPs recorded at the indicated sites (mPFC in blue; NAc in magenta; CA1 in brown; MS in yellow; and MD in cyan) from WT and PVKO mice. Illustrated records were collected from one second before to one second after crossing (dotted line) the light beam when approaching the lever. (**B**) Representative LFPs collected when mice pressed the lever (double arrowed line). **C** Representative LFPs collected when crossing the light beam to reach the feeder (dotted line). Calibrations in (**C**) are same for (**A**) and (**B**). Significant differences for the indicated frequency bands ($\delta$: 1–4 Hz; $\theta$: 4–12 Hz; $\beta$: 12–30 Hz; low$-\gamma$: 30–50 Hz; and high$-\gamma$: 50–150 Hz) were calculated from 16 similar LFP segments collected from n = 4 mice. Note the significant differences (* $P < 0.05$; **$P < 0.01$; ***$P < 0.001$; significances are indicated in color intensity for each selected brain site) between LFP spectral powers obtained from the two groups of mice during these appetitive (**A**,**B**) and consummatory (**C**) behaviors. The symbol ${\diamondsuit }$ indicates the frequency bands in which the LFP spectral powers from PVKO mice were significantly decreased with respect to those from WT mice (for details see Supplementary Table S2).

In the present task, the mice had to approach the lever (*“going-to-the-lever”*) and to press it (*“pressing-the-lever”*). Immediately after, the animal had to move towards the next corridor, approach the feeder (*“going-to-the-feeder”*) and pick the pellet (Fig. 2A–C). Animal approaching to the lever and feeder were detected by light beam located in the corresponding corridor, while lever press was detected by its activation. During the 10 sessions of the light/dark conditioning task LFPs were recorded in mPFC, NAc, CA1, MS, and MD. In only n=4 mice in each group, we could record simultaneously good quality LFP traces from all the electrodes implanted in the targeted regions.

Single channel recordings in targeted areas from the remaining animals were preserved for other analyses. Representative samples of LFPs, recorded from the five brain sites during the selected behavioral activities, are illustrated in Fig. 2A–C. A quantitative analysis of 16 similar LFP segments/brain site collected from n = 4 mice indicated that, in general, LFPs recorded from PVKO mice were flatter and less structured than those collected from WT controls, mainly those recorded from mPFC, NAc and CA1. In the spectral analysis, we considered the following five frequency bands, i.e. $\delta$ (1–4 Hz), $\theta$ (4–12 Hz), $\beta$ (12–30 Hz), low-$\gamma$ (30–50 Hz) and high-$\gamma$ (50–150 Hz).

The most significant differences (by ANOVA estimate of variance with $F_{(1,30)} > 10.0$) of spectral powers between WT and PVKO mice were observed at $\gamma$ bands in mPFC, at high-$\gamma$ band in NAc and at all frequency bands in CA1, irrespective of the three behavioral stages examined here (Fig. 2A–C and Supplementary Table S2). On the contrary, only at stage *“pressing-the-lever”* we observed significant differences in power spectra recorded in MS (in particular at $\theta$ band). In MD, the most significant differences were also observed at stage *“pressing-the-lever”*, at $\beta$ band low-$\gamma$ bands (Fig. 2A–C and Supplementary Table S2).

### Cortical LFPs collected from PVKO mice present selective differences from those collected from WT animals during the correct or incorrect performance of the operant conditioning task

**Figure 3:**
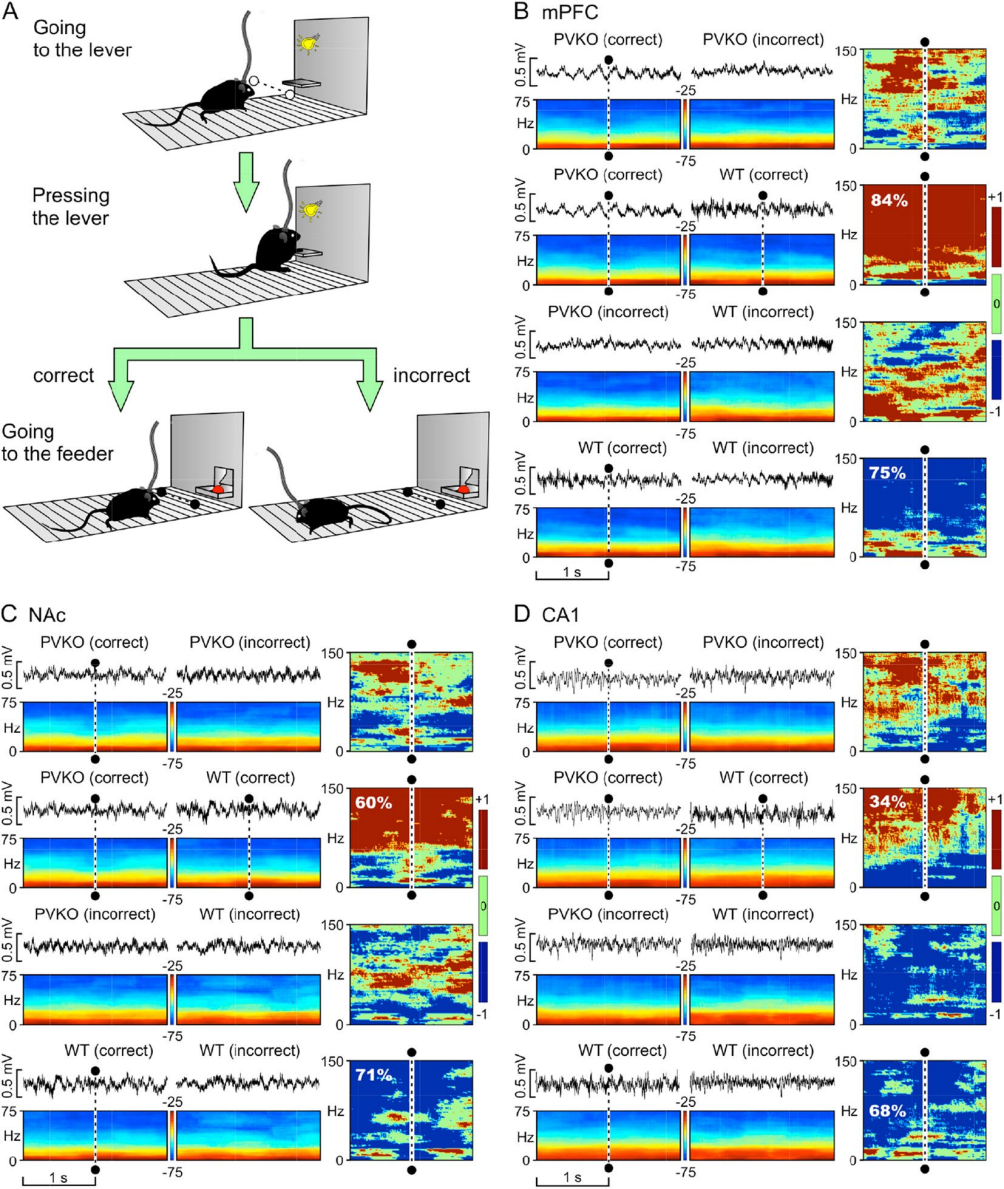
Differences in LFPs recorded at mPFC, NAc and CA1 from WT and PVKO mice during the correct or incorrect outcome of the operant conditioning trial. (**A**) Comparison of LFPs recorded when mice, after pressing the lever, approached (correct behavior) or not (incorrect behavior) the feeder. (**B**) Comparison of the dynamic changes in LFP recorded at the mPFC in WT and PVKO mice during the correct or incorrect conclusion of the operant conditioning task. From top to bottom are illustrated four different comparisons corresponding to WT and PVKO mice and to the correct or incorrect conclusion of the task. Within each panel are illustrated the selected animal group and behavior and a representative example of the recorded LFP activity corresponding to the same time window. An average of 8 samples: time/frequency representation of this LFP activity is illustrated below (frequency range, 0–75 Hz). When present, the dashed line indicates the moment of light beam crossing (black dots and dashed line). At the right of each panel is shown the probabilistic map (frequency range, 0–150 Hz) corresponding to the comparison of the two spectrograms located at the left. In these probabilistic maps, the blue [inference type − 1; the power in first (most leftward) spectrogram $\gg$ the power in second (rightward) spectrogram]  and brown [inference type + 1; the power in first (most leftward) spectrogram $\ll$ the power in second (rightward) spectrogram]  colors denote significant ($P < 0.05$; jackknifed estimates of the variance) statistical differences, while the green color (inference type 0; the power in first spectrogram $\approx$ the power in second spectrogram) indicates no significant ($P > 0.05$) differences. (**C**) Comparison for LFPs recorded in the NAc, as presented in (**B**). (**D**) Comparison for LFPs recorded in the hippocampal CA1 area, as presented in panel (**B**). For more details see Supplementary Table S3.

In several occurrences, mice of both groups did not complete the sequence—namely, they approached the lever and pressed it, but did not go to the feeder to collect the pellet. In case the mice did not go to the feeder, the trials were labeled as incorrect (Fig. 3A). We carried out a comparative analysis of LFPs collected from the five selected brain sites triggered by the light beam crossing when approaching the feeder (see dashed lines in recordings and spectrograms illustrated in Fig. 3B–D) *vs.* those trials when the animal did not approach the feeder after pressing the lever (unlabeled recordings and spectrograms illustrated in Fig. 3B–D). A systematic comparison of spectrograms (panels in the rightmost columns of Fig. 3B–D) during correct or incorrect behavior showed significant differences between the mice groups (see Supplementary Table S3).

During correct trials, the comparison of power spectra of WT and PVKO showed that in mPFC, NAc and CA1 of WT mice the power of $\beta$ and $\gamma$ bands were larger than in PVKO (second rows of panels in Fig. 3B–D; i.e., [high density of *P*-values < 0.05 in brown, inference type + 1, $E_{2nd}$ (the estimate of power in the second spectrogram) $\gg$ $E_{1st}$ (the estimate of power in the first spectrogram), jackknifed estimates of the variance ]). The corresponding probability densities (inference type + 1) were of 84% (mPFC), 60% (NAc) and 34% (CA1). In WT mice, the comparison of correct *vs.* incorrect trials (fourth rows of panels in Fig. 3B–D) showed that correct trials were characterized by larger power of $\beta$ and $\gamma$ bands than incorrect trials (i.e., [high density of *P*-values $< 0.05$ in blue, inference type – 1, $E_{1st}$ (the estimate of power in the first spectrogram) $\gg$ $E_{2nd}$ (the estimate of power in the second spectrogram), jackknifed estimates of the variance]). The statistical criterium set to determine the occurrence of a significant difference between WT and PVKO was that the condition 1 (inference type ±1 representing more than of 56.7% of the probability density) and condition 2 (inference type 0 representing less than of 33.3% of the probability density) were simultaneously verified. The corresponding probability densities (inference type –1,) were of 75% (mPFC), 71% (NAc) and 68% (CA1) (left panels in fourth rows of Fig. 3B–D). In PVKO mice, the comparison of power spectra during correct *vs.* incorrect trials (first rows of Fig. 3B–D) showed that the criterion for a significant difference in the probability density maps was reached only in MD (61% of inference type –1 and 24% of inference type 0; see Supplementary Table S3, Figure not shown). In MS, as expected from looking at Fig. 2C and from a systematic comparison of frequency bands in the three behavioral stages (Supplementary Table S2), the criterium was not verified for any of the probabilistic maps. For more details on this analysis, see Supplementary Table S3.

**Figure 4:**
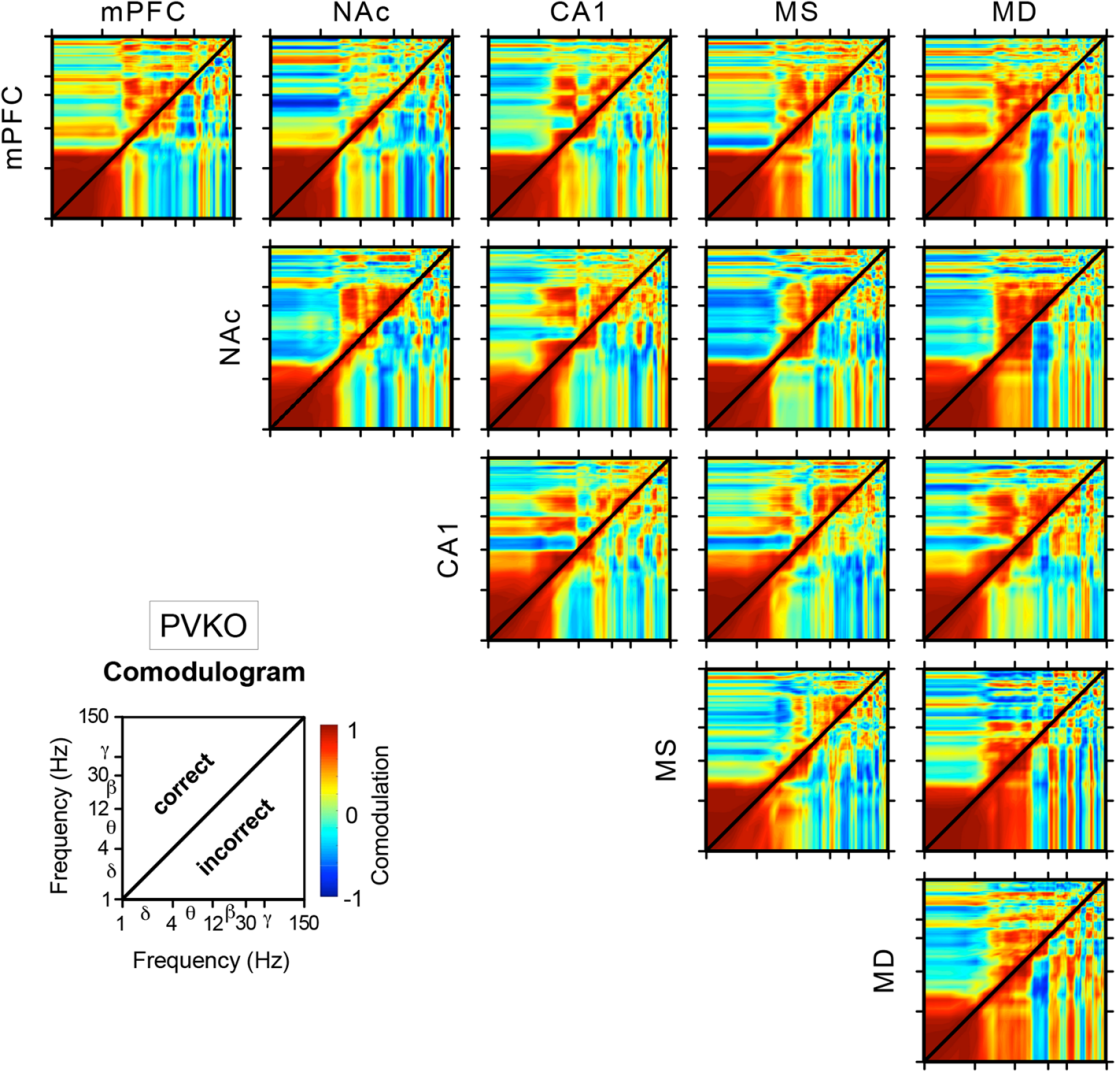
Power-power cross-frequency comodulagrams, in $\mathrm {log}_{10}-\mathrm {log}_{10}$ scale, of PVKO mice. Comodulogram (see Eq. 2 in “Materials and methods”) maps show coupling between pairs of LFP spectra collected from mPFC, NAc, CA1, MS and MD of PVKO mice after correct (upper triangular map) and incorrect (lower triangular map) outcome of the operant task. Notice that non-symmetric patterns indicate that correctness of the trial outcome influenced the cross-frequency coupling.

**Figure 5:**
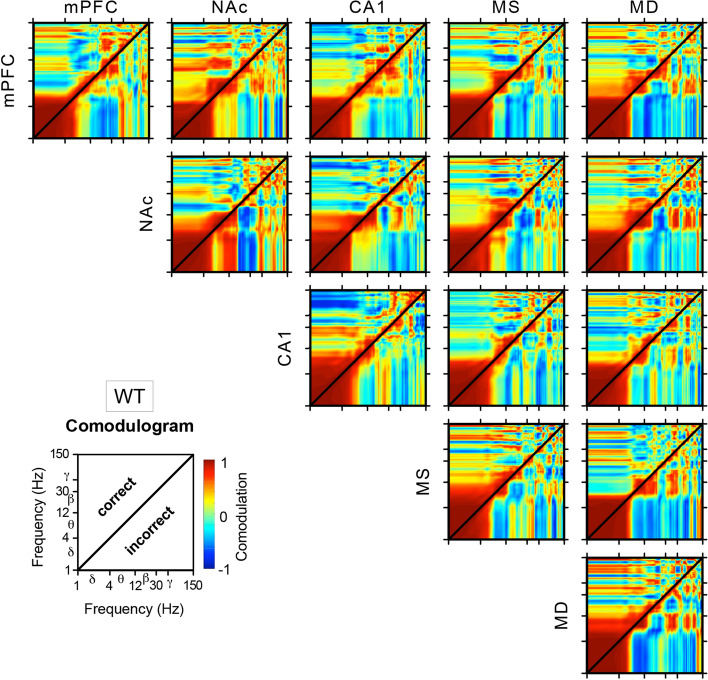
Power-power cross-frequency comodulagrams, in $\mathrm {log}_{10}-\mathrm {log}_{10}$ scale, of WT mice. Comodulogram (see Eq. 2 in “Materials and methods”) maps show coupling between pairs of LFP spectra collected from mPFC, NAc, CA1, MS and MD of WT mice after correct (upper triangular map) and incorrect (lower triangular map) outcome of the operant task. Notice that non-symmetric patterns indicate that correctness of the trial outcome influenced the cross-frequency coupling.

A cross-frequency coupling analysis, between pairs of LFPs across all channels recorded simultaneously, was carried out with a comodulation analysis (see Eq. 2 in “Materials and methods”) for PVKO and WT groups during correct and incorrect trials (Figs. 4, 5). The aim of this analysis is to investigate more in detail which frequency components might be affected by trial correctness in both strains of mice. In PVKO, the same-channel analysis (diagonal panels in Fig. 4) showed that in NAc $\times$ NAc we observed a positive comodulation during correct trials, but a negative or zero comodulation during incorrect trials in the [${\theta }-{\beta }$] $\times$ [${\beta }-\mathrm {low}{\gamma }$] frequency bands. On the contrary, in WT, the same-channel analysis (diagonal panels in Fig. 5) showed that the largest differences between correct and incorrect trials were observed in the MS $\times$ MS and MD $\times$ MD comodulograms, both characterized by a positive [${\delta }$] $\times$ [${\theta }$] comodulation pattern during correct trials and negative comodulation in this frequency band during incorrect trials.

The comodulation analysis across channels showed a similarity in the thalamo-cortical pattern of PVKO and WT in MD $\times$ mPFC with a strong negative comodulation in [${\beta }$] $\times$ [${\delta }-{\beta }$] frequency bands during incorrect trials. In both strains, the thalamo-cortical pattern showed a positive [${\beta }$] $\times$ [${\beta }$] comodulation during correct trials. The functional interaction between MD and NAc followed a similar picture for both strains, although to a lesser magnitude. It is worth noting that during correct trials we observed that comodulation between mPFC and NAc in the [${\beta }$] $\times$ [${\delta }-{\theta }$] frequency bands was negative in PVKO (Fig. 4) but positive in WT (Fig. 5). In PVKO during correct trials, we noticed also a positive comodulation in the frequencies [${\theta }$] $\times$ [${\beta }-\mathrm {low}{\gamma }$] between CA1 and all other channels. This pattern was absent in WT.

### Different dynamics of LFPs power spectra collected from mPFC, NAc and CA1 during the same session and across sessions in PVKO and WT mice

**Figure 6:**
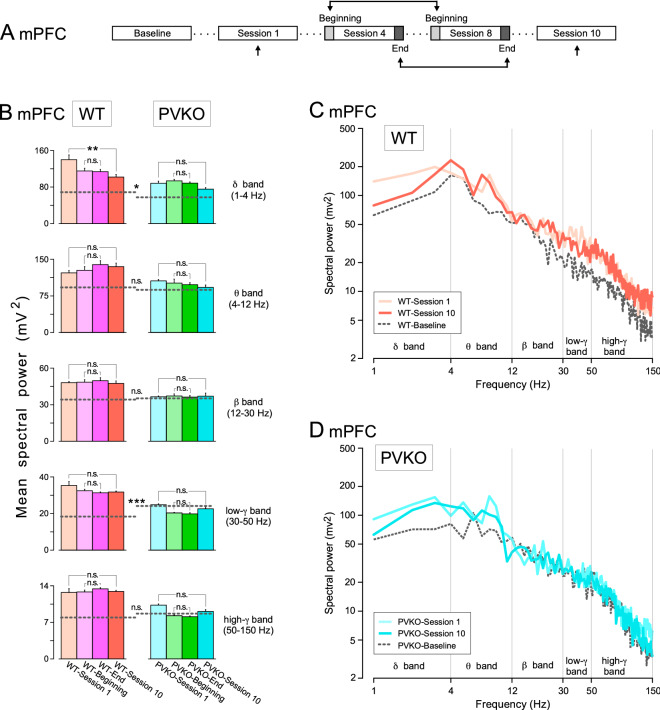
Spectral analyses of LFPs recorded in the mPFC across the successive operant conditioning sessions. (**A**) Experimental design. (**B**) Power histograms for the five selected frequency bands $\delta$ (1–4 Hz), $\theta$ (4–12 Hz), $\beta$ (12–35 Hz), low$-\gamma$ (30–50 Hz) and high$-\gamma$ (50–100 Hz) of LFPs recorded in the mPFC from WT (orange and pink) and PVKO (blue and green bars) mice *during the 1st session*, *at the beginning of the 4th–8th sessions*, *at the end of the 4th–8th sessions* and *during the 10th sessions*. Differences between the spectral powers in the selected frequency bands are indicated at the enclosed histograms for both WT and PVKO mice. The dotted lines across the histograms of each frequency band correspond to the mean power of the *baseline condition* for the corresponding group of mice. Note the larger spectral powers presented by LFPs collected from WT mice for the five selected frequency bands. Tukey-Kramer multiple comparisons test: n.s., non-significant difference; *$P < 0.05$; **$P < 0.01$; ***$P < 0.001$ (see Supplementary Tables S4.1–S4.3). (**C**) Mean power spectra of LFPs recorded in the mPFC from WT mice *during the baseline condition* and *during the 1st session* (light orange trace) and *during the 10th session* (dark orange trace). (**D**) Mean power spectra of LFPs recorded in the mPFC from PVKO mice *during the baseline condition* and *during the 1st session* (light blue trace) and *during the 10th session* (dark blue trace).

**Figure 7:**
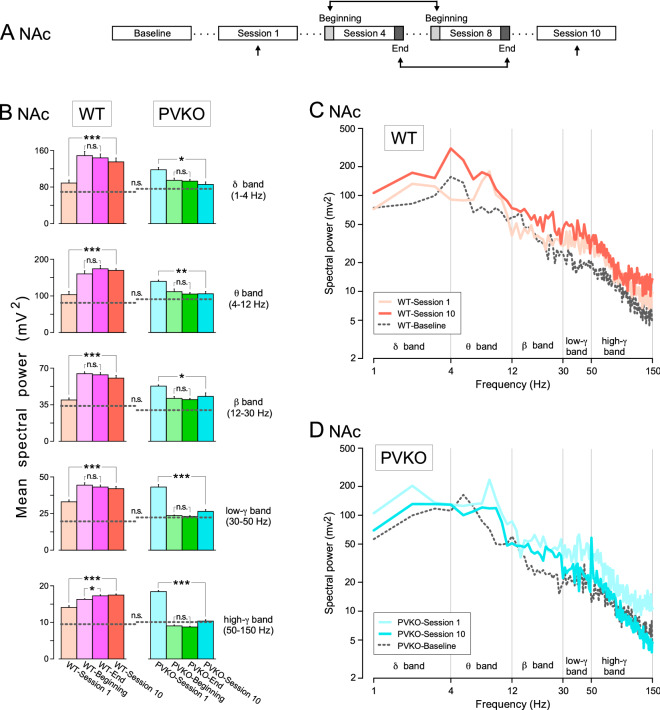
Spectral analyses of LFPs recorded in the NAc across the successive operant conditioning sessions. (**A**) Experimental design. (**B**) Power histograms for the five selected frequency bands of LFPs recorded in the NAc from WT (orange and pink) and PVKO (blue and green bars) mice *during the 1st session*, *at the beginning of the 4th–8th sessions*, *at the end of the 4th–8th sessions* and *during the 10th sessions*. Differences between the spectral powers in the selected frequency bands are indicated at the enclosed histograms for both WT and PVKO mice. The dotted lines across the histograms of each frequency band correspond to the mean power of the *baseline condition* for the corresponding group of mice. Note the larger spectral powers presented by LFPs collected from WT mice for the five selected frequency bands. Tukey-Kramer multiple comparisons test: n.s., non-significant difference; *$P < 0.05$; **$P < 0.01$; ***$P < 0.001$ (for details see Supplementary Tables S5.1-S5.3). (**C**) Mean power spectra of LFPs recorded in the NAc from WT mice *during the baseline condition* and *during the 1st session* (light orange trace) and *during the 10th session* (dark orange trace). (**D**) Mean power spectra of LFPs recorded in the NAc from PVKO mice *during the baseline condition* and *during the 1st session* (light blue trace) and *during the 10th session* (dark blue trace).

**Figure 8:**
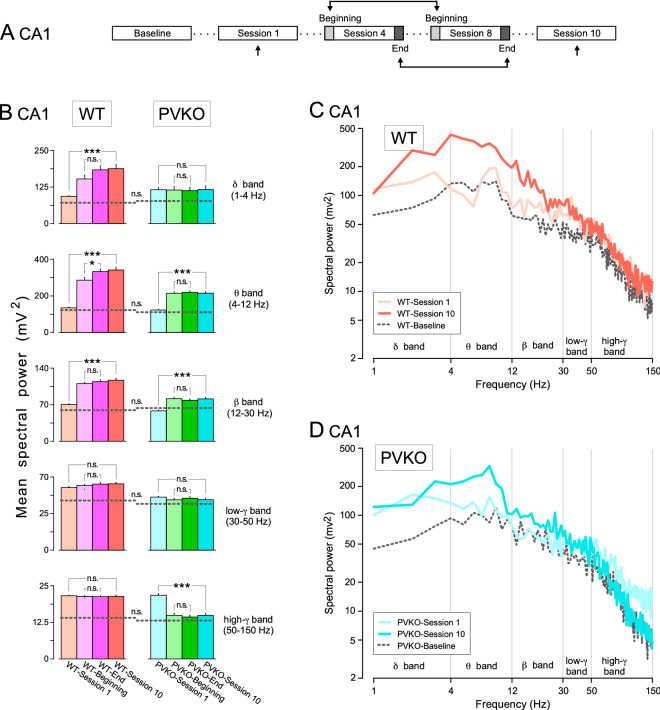
Spectral analyses of LFPs recorded in the hippocampal CA1 area across the successive operant conditioning sessions. (**A**) Experimental design. (**B**) Power histograms for the five selected frequency bands of LFPs recorded in the CA1 from WT (orange and pink) and PVKO (blue and green bars) mice *during the 1st session*, *at the beginning of the 4th–8th sessions*, *at the end of the 4th–8th sessions* and *during the 10th sessions*. Differences between the spectral powers in the selected frequency bands are indicated at the enclosed histograms for both WT and PVKO mice. The dotted lines across the histograms of each frequency band correspond to the mean power of the *baseline condition* for the corresponding group of mice. Note the larger spectral powers presented by LFPs collected from WT mice for the five selected frequency bands. Tukey-Kramer multiple comparisons test: n.s., non-significant difference; *$P < 0.05$; **$P < 0.01$; ***$P < 0.001$ (for details see Supplementary Tables S6.1–S6.3). (**C**) Mean power spectra of LFPs recorded in the CA1 from WT mice *during the baseline condition* and *during the 1st session* (light orange trace) and *during the 10th session* (dark orange trace). (**D**) Mean power spectra of LFPs recorded in the CA1 from PVKO mice *during the baseline condition* and *during the 1st session* (light blue trace) and *during the 10th session* (dark blue trace).

The quantitative assessment of the effect of operant conditioning on the power spectra of LFPs could be done during the same session (i.e., from its beginning to its end) and across sessions (i.e., from the 1st to the 10th) (Figs. 6A, 7A, and 8A). We decided to consider *session 1* as representative of kickoff learning and *sessions 4 to 8* as representative of task acquisition. More specifically, we quantified the comparison of the power spectra of LFPs recorded during the first two minutes (*WT-Beginning* and *PVKO-Beginning*) with the last two minutes (*WT-End* and *PVKO-End*) of sessions 4-8 (i.e., merging five sessions overall). At *session 10* the animals achieved the criteria set to determine that operant conditioning was acquired. Hence, the rationale was to determine the dynamics of LFPs power spectra along different phases of learning the operant conditioning and compare PVKO with WT. We focused our analysis on mPFC, NAc and CA1 because these areas matched two statistical criteria (Supplementary Table S3), as mentioned above.

During the baseline condition (defined in “Materials and methods”), the spectral powers of LFPs of WT and PVKO did not show any significant difference at the five selected frequency bands in NAc and dorsal hippocampus CA1 area (see the Tukey-Kramer multiple comparison analyses in Supplementary Tables S5.1 and S6.1, respectively). At mPFC, we observed that power spectra of PVKO baseline was lower than WT baseline for the $\delta$ band (1-4 Hz) and larger than WT baseline for the low-$\gamma$ band (30-50 Hz) (Fig. 6B, Supplementary Table S4.1). For the comparison between all sessions at mPFC, we also observed that the spectral power of the five selected frequency bands was significantly larger in WT mice than in their littermate PVKO ones (Supplementary Tables S4.2, S4.3). It is interesting to notice that only the power spectra in the $\delta$ band collected from WT mice was significantly decreased ($P < 0.01$) from the 1st to the 10th session (Fig. 6B,C). Changes in the spectral power of the $\delta$ band have been related to the performance during the behavioral sequence from pressing the lever until eating the pellet^29^. In the remaining band frequencies of WT mice and in all band frequencies of PVKO, we observed that operant conditioning did not affect the spectral power at mPFC throughout the acquisition stages.

At the level of NAc, we observed that the acquisition of operant conditioning affected significantly the spectral power in all frequency bands from *session 1* to *session 10* in both WT and PVKO mice (Fig. 7B–D; Supplementary Table S5.3). However, a crucial difference between strains was that throughout the acquisition stages spectral power in NAc of WT increased (Fig. 7B,C), but spectral power in NAc of PVKO decreased (Fig. 7B,D). Except for a small increase between *WT-Beginning* and *WT-End* for the spectral power in the high-$\gamma$ band (50-150 Hz), no significant differences were observed from the beginning to the end of the same session, for sessions 4-8, in both WT and PVKO mice (Fig. 7B; Supplementary Table S5.2).

At the level of the CA1 area of the hippocampus, the most interesting observation was the similar pattern of spectral power increase induced by learning of operant conditioning from *session 1* to *session 10* in both WT and PVKO strains for the 4–30 Hz frequency range (i.e., for $\theta$ and $\beta$ bands, Fig. 8B–D; Supplementary Table S6.3). For the $\delta$ band, CA1 spectral power increased throughout the sessions of WT, but remained unaffected in PVKO mice. For the $\gamma$ bands, CA1 spectral power tended to stay unaffected in both WT and PVKO strains, apart from the *session 1* of PVKO, which was characterized by a large power in the high-$\gamma$ frequency range. We noticed also marginal differences, if any, for all frequency bands between *WT-Beginning* *vs.* *WT-End* and *PVKO-Beginning* *vs.* *PVKO-End* (Supplementary Table S6.2).

## Discussion

Mice performances in executive functions show strong similarities with humans^30^. In this study, we have presented evidence for the first time the ability of PV-deficient mice^1,2^ to learn an operant conditioning task with performance levels similar to controls, but following a delayed learning. Cognitive deficits in mice associated with altered function of fast-spiking PV positive interneurons were similar to impaired associative and outcome expectant function observed in humans with psychiatric disorders associated with alterations in the expression of PV, such as Tourette syndrome^31^, schizophrenia and bipolar disorders^32^, autism spectrum disorders^33^, and Huntington disease^34^. The acquisition curves of WT mice during the instrumental learning task were similar to controls of the same species reported in previous studies^20,21^. The task was acquired by PVKO mice, despite reaching smaller light/dark coefficients than WT (Fig. 1F). In our initial training, all mice had to reach the reward criterion pressing the lever at least 20 times per session for two successive sessions lasting 20 minutes each. Hence, differences in the total amount of responses per session could represent a serious confound, as one group would have experienced fewer trials during which to reach the criterion. During the acquisition of the operant training, differences in the dynamics of learning between PVKO and WT were particularly emphasized during sessions 3, 4 and 5 (Supplementary Tables S1.1, S1.2). The locomotor pattern of PVKO mice is characterized by increased average activity, with movements faster than controls, but less motor coordination^10^. Due to the complex differences in locomotor pattern between WT and PVKO, it is difficult to state whether other criteria, e.g. the fixation of a number of awarded trials per session during two (or three) successive sessions and/or the fixation of a maximum latency to get the reinforcement, could constitute a better measurement to assess the achievement of initial associative learning prior to true operant training. We decided to stick to the same criterion set in our laboratory for a series a previous studies for the sake of comparison –namely, the number of lever presses carried out during the light and dark periods^20,21,29,35^. Extended behavioral studies with several variants of the protocol should be considered in the future for a careful evaluation and separation of the behavioral responses associated with motivation from locomotion and motor coordination.

It was demonstrated that during tasks demanding attention, the activity of prefrontal PV-expressing interneurons is modified, with the activity being higher and in the gamma range (30-40 Hz) during attentional processing resulting in successful execution of a task^36^. A detailed analysis of LFPs simultaneously recorded in selected structures classically related to operant conditioning tasks has been carried out here in order to properly determine putative functional deficits associated to specific phases of the task. In PVKO mice we observed a decreased signal-to-noise ratio in LFP recordings during conditioning, likely to be associated with a tendency to higher firing rates observed in several brain areas of PVKO in single unit recordings^4,7,8,37^. This result could be due to a differential recruitment of PV-positive GABAergic neurons during the execution of the task modulated by the balance of excitation and inhibition (E/I)^38^, thus controlling and tuning the transitions between oscillatory brain states^39^. PV-expressing interneurons in mPFC could fire in millisecond synchrony and exert a rapid and powerful inhibition on the activation of the pyramidal cells, thus controlling the outputs of this cortical area^40^. However, whether mutual inhibition alone between GABAergic cells, or whether re-entrant excitation from recurrent networks of inhibitory and excitatory neurons, is necessary to develop high-frequency oscillations remains an unresolved question, because both mechanisms exist^41^.

Our LFPs recordings showed similar spectral power at all frequency bands during baseline condition, but significant decreases of the spectral power in $\beta$ - and $\gamma$-bands in PVKO compared with WT mice across conditioning sessions and, particularly, during approaching-the-lever-and-feeder sequence. It is known that the plasticity of PV basket cells play a key role in rule learning^42^ and in mice, during a reward foraging task, these cells responded at reward leaving and encoded preceding stay duration, but did not respond to reward approach^40^. The activity of fast-spiking GABAergic PV-expressing interneurons is directly linked to oscillations in the $\gamma$-band and the modulation of neuronal rhythms during cognitive engagement^9,43^. The spectral power comodulation analysis presented here showed interesting differences between correct and incorrect trials, but similar patterns in both PVKO and WT mice with respect to thalamo-cortical and thalamo-NAc comodulograms. On the contrary, during correct trials, the comodulation between mPFC and NAc in the [${\beta }$] $\times$ [${\delta }-{\theta }$] frequency bands was negative in PVKO but positive in WT mice. This finding appears in agreement with the literature showing that avoidance behavior in a real-time place preference task could be provoked by the optogenetic stimulation of corticofugal long-range GABAergic projections of PV-positive interneurons to the nucleus accumbens^44^. Moreover during correct trials, we noticed only in PVKO mice and not in WT a positive comodulation between CA1 and all other channels in the frequencies [${\theta }$] $\times$ [${\beta }-\mathrm {low}{\gamma }$]. This is in agreement with a previous study of the non-linear coupling of LFPs in the cerebral cortex of PVKO mice^45^ showing an abnormally high proportion of coupling in the ${\theta }$-${\beta }$ range that suggested interactions at longer range than in WT mice. This pattern was completely opposite to that recognized in WT mice, in which their LFPs showed mainly low-$\gamma$ frequency (30-50 Hz) components^45,46^.

A critical part of the associative learning process in operant conditioning, by which the brain links natural rewards or alternative rewards, such as drug or alcohol, with specific stimuli and motor response, is encoded in the nucleus accumbens^47,48^ and expressed by $\gamma$ oscillations^49,50^. It is of particular interest our observation of how operant conditioning modified the spectral power for different frequency bands in the NAc throughout the learning process. From session 1 to session 10, the spectral power increased in WT and decreased in PVKO mice. It is also worth noting that in PVKO no differences were observed in spectral power between *PVKO-Beginning* and *PVKO-End* and between these measurements and powers at session 10. This significant set of changes is likely to be related with the increase contribution of food reward across training, a typical characteristic of NAc neurons^51^ and with some but not all neurophysiological deficits seen in GABAergic interneurons of PV-deficient mice^52^. During social interaction in normal conditions, the increase of the neurotransmitter DA in the NAc would excite the PV-expressing fast-spiking interneurons (PV-FSI) directly through D1-type receptors or indirectly through D2-type receptors^53^, and cortical projections from mPFC to NAc would contribute to maintaining the E/I balance and regulating reward-seeking processes^28^. This would increase the inhibitory activity of the PV-FSI on the medium spiny neurons (MSN), which would generate oscillations in $\gamma$-frequency range. Conversely, stress-induced rats by social interaction with unfamiliar conspecific exhibited decreased $\gamma$ oscillations in the NAc, as a consequence of a decreasing in the levels of DA in the NAc which provoked a decrease in the activity of the PV-FSI^54,55^. This might also suggest that PVKO mice are likely to be characterized by a reduced sensitivity to reward state, but our baseline condition could not reveal it because mice were left alone during LFP recordings. Both WT and PVKO strains could learn the operant conditioning task, but the differences observed in NAc LFPs suggest that the value of the reward was not encoded via the same mechanisms. Other interneurons subtypes are involved as well in the circuitry^56,57^, and altogether they may make an asymmetric contribution to regulate not only high-frequency, but also low-frequency oscillatory activities.

The activity of fast-spiking PV-expressing interneurons in hippocampal microcircuits is thought to play a key role in network oscillations^58^. High-frequency oscillations in the ranges of the $\beta$ - and $\gamma$-bands (30–80 Hz) have been suggested to represent a fundamental mechanism of information processing in the forebrain^59^, although this interpretation is challenged by new observations^60,61^. Hippocampal activity in behaving mice during exploration is characterized by $\theta$ (6–9 Hz) and $\gamma$ (30–100 Hz) oscillations^54^. Gamma power and theta power of LFPs in CA1 hippocampal area were comodulated and $\gamma$ power varied as a function of the $\theta$ cycle^62^. In this study, we observed a positive comodulation of power between CA1 and all other channels only during correct trials by PVKO mice in the bands [${\theta }$] $\times$ [${\beta }-\mathrm {low}{\gamma }$]. However, this pattern was absent in WT mice. This may suggest that the hippocampal activity pattern of PVKO animals is somehow staying in an exploratory mode triggered by the correctness of the trial. The dynamics of the spectral power throughout the operant conditioning showed an increase between the first and the last session in the ${\theta }$ and ${\beta }$ frequency bands of both WT and PVKO strains. This finding suggests the uncoupling of CA1 spectral power and its comodulation along the learning stages. It is interesting that in PVKO mice usual high-$\gamma$ LFP patterns restricted to CA1^62^ decreased spectral power after session 1 and stayed low till the last session of recording.

In conclusion, the current results suggest that the modifications of the LFP activities observed in PV-deficient mice during operant conditioning were consistent with changes in network dynamics associated with a modified encoding of reward value in the nucleus accumbens. These alterations compromised specific brain oscillations in basal ganglia-thalamo-cortical circuit recorded here throughout the operant conditioning task and also during the execution of a precise behavioral sequence (from pressing the lever until eating the pellet).

## Materials and methods

### Animals

PVKO mice (strain name: B6.$\hbox {Pvalb}^{tm1Swal}$) were generated in the laboratory of Prof. Beat Schwaller (Anatomy Unit, Department of Medicine, University of Fribourg, Switzerland) by homologous recombination^2^. Congenic C57Bl/6J mice could be used as control wild-type (WT) animals^63^. Only male animals were used in this study. Upon arrival of the PVKO mice and their littermate control to the Animal Facilities of Pablo de Olavide University, the animals were placed in individual cages provided of an enriched environment and of nesting materials. Animals were maintained in the same situation until the end of the experiments. They were provided with food and water ad libitum and maintained in a temperature-controlled environment in a 12/12 h light-dark cycle. All behavioral experiments were performed in accordance with European Union Council (2010/276:33-79/EU) guidelines and Spanish (BOE 34:11370-421, 2013) regulations for the use of laboratory animals in chronic experiments. Experiments were also approved by the local Ethics Committee of the Pablo de Olavide University.

### Surgery

Animals were anesthetized with 0.8–1.5 % isofluorane (Astra Zeneca, Madrid, Spain) delivered from a calibrated mask (Cibertec, Madrid, Spain). Once anesthetized, and following surgical procedures described elesewhere^64^, animals were chronically implanted with five recording electrodes aimed at the following target areas^65^: mPFC (anteroposterior, AP: 2; lateral, L: 0.3; depth from brain surface, D: 1.6); NAc (AP: 1.25; L: 1.1; D: 3.75); CA1 (AP: 2.2; L: 1.2; D:1.3); MS (AP: 0.5; L: 0.1; D: 3.8); and, MD nucleus (AP: 1.75; L: -0.4; D: 3.2) (Fig. 1A,B). Electrodes were made of 50 $\mu$m Teflon-coated tungsten wire $14-16~k{\Omega }$ of impedance; Advent Research Materials, Eynsham, UK). Two bare silver wires (0.1 mm in diameter) were affixed to the skull as ground. Electrodes were connected to two 4-pin sockets (RS-Amidata, Madrid, Spain). Sockets were fixed to the skull with the help of four small screws and dental cement. Animals were allowed a week to recover before the start of the recording sessions.

### Operant conditioning

Operant conditioning took place in two Skinner box modules ($12.5 \times 13.5 \times 18.5$ cm; from Med Associates, St. Albans, VT 05454, USA). Each Skinner box was housed within a home-made, sound-attenuating chamber ($90 \times 55 \times 60$ cm), constantly illuminated (19 W lamp) and exposed to a 45 dB white noise (Cibertec). Skinner boxes were equipped with a food dispenser from which pellets (MLabRodent Tablet, 20 mg; Test Diet, St. Louis, MO 63144, USA) could be delivered by pressing a metal lever. Each Skinner box was provided with a division wall (Fig. 1C) located between lever and feeder modules, and with two light beams placed at 6 cm of the lever and the feeder, respectively^35^.

Before training, mice were handled daily for 7 days and food deprived to 90% of their free-feeding weight. During the last 2 days of this phase, a *baseline* condition was defined as follows. The mice were left for 5 minutes in a methacrylate box ($20 \times 12 \times 15$ cm) without levers, no feeder, or food, with the electrodes connected for recording baseline LFPs. Following standard procedures of our laboratory^21^, animals were trained initially to press the lever to receive a pellet from the feeder using a fixed-ratio (1:1) schedule (Fig. 1C). Training sessions lasted for 20 minutes. Animals were maintained on this 1:1 schedule until they reached the selected criterion—namely to press the lever for at least 20 times along two successive sessions. All animals reached the selected criterion in > 5 training sessions. Once criterion for the 1:1 schedule was reached, the true operant training was carried out using a light/dark protocol (Fig. 1C). In this protocol, only lever presses performed by the experimental animal during the lighted period (20 s) were reinforced with a pellet. Lever presses performed during the dark protocol ($20 \pm 10$ s) were not reinforced. In addition, lever presses carried out during the dark period restarted the dark protocol for an additional random (1-10 s) time. LFPs were recorded with Grass P511 differential amplifiers (Grass Instruments, Astor-Med Inc., West Warwick, RI 2893; USA). All operant sessions were recorded with a video-capture system (Sony HDR-SR12E, Madrid, Spain) synchronized to LFP recordings.

### Histology

We apply classical techniques for checking the recording sites and for reconstruction of electrode penetrations, used for many years in our laboratory^21,35^. At the end of the experimental sessions, mice were deeply reanesthetized (sodium pentobarbital, 50 mg/kg) and perfused transcardially with saline and 4% phosphate-buffered paraformaldehyde. Their brains were removed, postfixed overnight at 4 °C, and cryoprotected in 30% sucrose in PBS. Sections were obtained in a microtome at 50 μm. Selected sections including brain implanted sites were mounted on gelatinized glass slides and stained using the Nissl technique with 0.1% toluidine blue to determine the location of recording electrodes (Fig. 1B).

### Statistics, data analyses and representations

LFP and 1.0 V rectangular pulses corresponding to lever presses, pellet delivery, and light-beam crossings were stored digitally on a computer through an analog-to-digital converter (CED 1401 Plus; Cambridge Electronics Design, Cambridge, UK). Data were analyzed offline for quantification of animal performance in the Skinner box (Fig. 1D) and LFPs with the Spike 2 (Cambridge Electronics Design) program and the video-capture system (Figs. 2–8). Statistical analyses were carried out using the Sigma Plot 11.0 package (Sigma Plot, San Jose, CA, USA) and the Statistics MATLAB Toolbox (version 9.4, R2018a. The MathWorks, Natick, MA, USA) for Windows, for a statistical significance level of $P < 0.05$. For multivariate statistics assessments, both parametric (Fisher ANOVA *F*-tests, without or with repeated measures) and non-parametric (ANOVA tests on ranks, without repeated measures (Kruskal-Wallis ANOVA)) methods were used to assess the statistical significance of differences between groups, followed by the appropriate test (Holm-Sidak, Tukey-Kramer, or Student–Newman–Keuls, in this order of priority) for all the pairwise multiple-comparison analyses^21,35^. Unless otherwise indicated, data are always represented as the mean± SEM. The performance of WT and PVKO mice during the light/dark test was assessed by mean of the light/dark (*LD*) coefficient defined as:

1 $$\begin{aligned} {LD}~~{coefficient} = (N_{lp,L} - N_{lp,D}) / N_{lp,T} \end{aligned}$$

where $N_{lp,L}$ and $N_{lp,D}$ are the number of lever presses during the lighted (*L*) and dark (*D*) periods, respectively; and $N_{lp,T}$ is the total (*T*) number of lever presses (Fig. 1F).

For LFP analysis, we used data collected from sessions presenting significantly different performance in the operant conditioning task. Following previous studies from our laboratory^21^, LFP epochs lasting 2 s each were collected during the performance of the following behaviors: (i) when crossing the light beam to reach the lever; (ii) when pressing the lever; and, (iii) when crossing the light beam to reach the feeder (Fig. 2). LFP epochs were also checked from the corresponding video recordings before their selection for LFP analysis^21^. Analytical procedures, including the frequency domain (using the fast Fourier transform) and the time-frequency (using the short-term Fourier transform) analyses of the LFP recordings, as well as the quantification and representation scripts, were developed in a previous study from our laboratory^21^ with the help of MATLAB routines and customized scripts of Chronux^66,67^ software (versions 2.11/R2014 and 2.12/R2018. Website: http://chronux.org/).

The time-frequency spectrograms enabled showing the frequency band of pure LFP signal components^68^, since they included an estimation of the short-term, time-localized frequency content of the signals (spectrograms in Fig. 3). Probability maps for the comparison of pairs of spectrograms were generated following previous descriptions by our group^35^. We selected the following frequency bands: delta ($\delta$), 1–4 Hz, theta ($\theta$), 4–12 Hz; beta ($\beta$), 12–30 Hz; low-gamma ($\gamma$), 30–50 Hz; and high-$\gamma$, 50–150 Hz. The algorithm included the analysis of mean values of the spectral powers between the different frequency bands inside each epoch, and the analysis of mean values of the spectral powers for the same frequency band between the different epochs^21^.

In this work, the following spectral measures were calculated to assess the spectral couplings between different oscillatory activities from LFP recordings:

2 $$\begin{aligned} \rho _{i,j} = \sum _{\Delta t_{\kappa }} \big ( SP_{\Delta t_{\kappa }} (f_{i}) - {\overline{SP}} (f_{i}) \big ) \big ( SP_{\Delta t_{\kappa }} (f_{j}) - {\overline{SP}} (f_{j}) \big ) / \sigma _{i} \sigma _{j} \end{aligned}$$

where $\rho _{i,j}$ is the cross-frequency comodulation^69,70^ (i.e., the Pearson product moment correlation of spectral magnitudes) between the spectral power *SP*(*f*) at a frequency $f_{i}$ and at another frequency $f_{j}$ within the same LFP spectrum. The term $SP_{\Delta t_{\kappa }} (f_{i})$ is the spectral power at frequency $f_{i}$ in time-window ${\Delta t_{\kappa }}$ and the term ${\overline{SP}} (f_{i})$ is the average spectral power magnitude at frequency $f_{i}$ over all time-windows ranging in *k*. The parameter $\sigma _{i}$ is the standard deviation of the spectral power at frequency $f_{i}$. Similar definitions were adopted for describing the above terms and parameters at frequency $f_{j}$. The term cross-frequency “comodulation” generally denotes the interaction between specific features of two oscillations, such as amplitude and phase. Here, we calculate the power-power comodulation^71,72^, which the most commonly used spectral measure of cross-frequency couplings between two oscillations. For more details see Figs. 4, 5.

To obtain the LFP spectrograms (see Fig. 3) and the LFP-LFP comodulagrams (see Figs. 4, 5) LFP spectra were normalized independently for each frequency bin as the Z score of the power for that bin. In addition, to carry out the multiple comparison analyses (Figs. 6–8) all the spectral power changes were referred to both the baseline condition without behavioral component (Supplementary Tables S4.1–S6.1) and the experimental condition in which the animals were performing the behavioral task in the Skinner box (Supplementary Tables S4.2–S6.2 and S4.3–S6.3).

## Supplementary Information

Supplementary Tables.

## Abbreviations

PVKO: Parvalbumin-knockout
WT: Wild type
mPFC: Medial prefrontal cortex
MD: Mediodorsal thalamic nucleus
MS: Medial septum
NAc: Nucleus accumbens
CA1: Hippocampal CA1 area
LFPs: Local field potentials
Delta: ($$\delta$$)
Theta: ($$\theta$$)
Beta: ($$\beta$$)
Gamma: ($$\gamma$$)
