# Supplementary material for: Operant conditioning deficits and modified local field potential activities in parvalbumin-deficient mice
Source: Sci Rep. 2021 Feb 3;11:2970. doi: 10.1038/s41598-021-82519-3 (PMC7859233; doi:10.1038/s41598-021-82519-3)
Supplement: Supplementary file 1 — Supplementary Tables. [file 41598_2021_82519_MOESM1_ESM.pdf]

Supplementary Material

Supplementary Tables (13): S1.1–S1.2, S2, S3, S4.1–S4.3, S5.1–S5.3, S6.1–S6.3

Operant conditioning deficits and modified local field potential activities in parvalbumin-deficient mice

A. Lintas[1*], R. Sánchez-Campusano[2], A.E.P. Villa[1], A. Gruart[2], J.M. Delgado-Garcia[2]

[1]University of Lausanne, Lausanne, Switzerland

[2]Pablo de Olavide University, Seville, Spain

Table S1.1. Statistical reports corresponding to the dynamic evolution of the light/dark coefficient (see Eq. 1 in Material and Methods) across conditioning sessions (see **Fig. 1F** in the main text).

**Multiple Comparisons for Factor (GROUP and GROUP within SESSION): Holm-Sidak method**						
**Comparison: WT vs. PVKO**		**WT Means**	**PVKO Means**	**Diff. of Means**	**| *t*-value |**	***P*-value**
Between Groups (WT vs. PVKO)		0.021	-0.307	0.328	3.349	0.010 *
Between Groups - Session 1		-0.681	-0.650	0.031	0.203	0.840 n.s.
Between Groups - Session 2		-0.537	-0.706	0.169	1.102	0.277 n.s.
Between Groups - Session 3		-0.305	-0.619	0.314	2.044	0.048 *
Between Groups - Session 4		0.095	-0.409	0.504	3.285	0.002 **
Between Groups - Session 5		0.183	-0.294	0.477	3.108	0.004 **
Between Groups - Session 6		0.098	-0.221	0.319	2.079	0.044 *
Between Groups - Session 7		0.140	-0.149	0.289	1.885	0.067 n.s.
Between Groups - Session 8		0.426	-0.064	0.490	3.192	0.003 **
Between Groups - Session 9		0.398	0.064	0.334	2.175	0.036 *
Between Groups - Session 10		0.389	-0.026	0.414	2.699	0.010 *
**Linear Regression [WT: $F_{(1,8)}$ = 46.413; $P < 0.001$]**		**Standard Error**			***t*-value**	***P*-value**
R^2 = 0.8530	Slope value = 0.1205	0.0177			6.8130	< 0.001
P = 0.0001	Constant = -0.6418	0.1100			-5.851	< 0.001
**Linear Regression [PVKO: $F_{(1,8)}$ = 118.565; $P < 0.001$]**		**Standard Error**			***t*-value**	***P*-value**
R^2 = 0.9368	Slope value = 0.0887	0.0081			10.8890	< 0.001
$P < 0.0001$	Constant = -0.7951	0.0505			-15.7340	< 0.001

Here, n.s. indicates non-significant differences; *, $P < 0.05$; **, $P < 0.01$; ***, $P < 0.001$. Yellow boxes highlight *t*-values larger than 3.

Table S1.2. Statistical reports corresponding to the dynamic evolution of the number of incorrect responses (lever presses in dark) between WT and PVKO mice across conditioning sessions.

**Multiple Comparisons for Factor (GROUP and GROUP within SESSION): Holm-Sidak method**					
**Comparison: WT vs. PVKO**	**WT Means**	**PVKO Means**	**Diff. of Means**	**| *t*-value |**	***P*-value**
Between Groups (WT vs. PVKO)	56.117	87.283	31.167	3.790	0.004 **
Between Groups - Session 1	168.000	129.667	38.333	1.929	0.057 n.s.
Between Groups - Session 2	119.333	150.667	31.333	1.577	0.118 n.s.
Between Groups - Session 3	73.167	135.333	62.167	3.128	0.002 **
Between Groups - Session 4	35.500	107.000	71.500	3.598	0.001 ***
Between Groups - Session 5	35.167	78.000	42.833	2.155	0.034 *
Between Groups - Session 6	33.667	66.667	33.000	1.661	0.100 n.s.
Between Groups - Session 7	30.833	66.500	35.667	1.795	0.076 n.s.
Between Groups - Session 8	21.833	52.833	31.000	1.560	0.122 n.s.
Between Groups - Session 9	23.667	41.667	18.000	0.906	0.367 n.s.
Between Groups - Session 10	20.000	44.500	24.500	1.233	0.221 n.s.

Here, n.s. indicates non-significant differences; *, $P < 0.05$; **, $P < 0.01$; ***, $P < 0.001$. Yellow boxes highlight *t*-values larger than 3.

Table S2. Statistical reports corresponding to the multiple comparisons for the mean values of the LFP spectral powers measured **from the five selected sites** (see **Fig. 2** in the main text).

**mPFC**	**WT vs. PVKO: LFP spectral powers measured from the medial prefrontal cortex (mPFC)**		
**Band**	**Fig. 2A: Going to the lever**	**Fig. 2B: Pressing the lever**	**Fig. 2C: Going to the feeder**
1-4 Hz	$F_{(1,30)}$ = 8.55; P = 0.007 ; **	$F_{(1,30)}$ = 0.22; P = 0.640 ; n.s.	$F_{(1,30)}$ = 0.06; P = 0.805 ; n.s.
4-12 Hz	$F_{(1,30)}$ = 0.00; P = 0.978 ; n.s.	$F_{(1,30)}$ = 0.29; P = 0.593 ; n.s.	$F_{(1,30)}$ = 0.67; P = 0.421 ; n.s
12-30 Hz	$F_{(1,30)}$ = 0.45; P = 0.508 ; n.s.	$F_{(1,30)}$ = 5.61; P = 0.025 ; *	$F_{(1,30)}$ = 6.80; P = 0.014 ; *
30-50 Hz	$F_{(1,30)}$ = 30.68; P < 0.001 ; ***	$F_{(1,30)}$ = 24.48; P < 0.001 ; ***	$F_{(1,30)}$ = 20.82; P < 0.001 ; ***
50-150 Hz	$F_{(1,30)}$ = 46.28; P < 0.001 ; ***	$F_{(1,30)}$ = 49.03; P < 0.001 ; ***	$F_{(1,30)}$ = 65.89; P < 0.001 ; ***
**NAc**	**WT vs. PVKO: LFP spectral powers measured from the nucleus accumbens (NAc)**		
**Band**	**Fig. 2A: Going to the lever**	**Fig. 2B: Pressing the lever**	**Fig. 2C: Going to the feeder**
1-4 Hz	$F_{(1,30)}$ = 1.75; P = 0.196 ; n.s.	$F_{(1,30)}$ = 0.77; P = 0.387 ; n.s.	$F_{(1,30)}$ = 4.25; P = 0.048 ; *
4-12 Hz	$F_{(1,30)}$ = 1.85; P = 0.184 ; n.s.	$F_{(1,30)}$ = 8.40; P = 0.007 ; **	$F_{(1,30)}$ = 1.32; P = 0.260 ; n.s.
12-30 Hz	$F_{(1,30)}$ = 6.71; P = 0.015 ; *	$F_{(1,30)}$ = 0.54; P = 0.467 ; n.s.	$F_{(1,30)}$ = 0.03; P = 0.861 ; n.s.
30-50 Hz	$F_{(1,30)}$ = 6.12; P = 0.019 ; *	$F_{(1,30)}$ = 0.85; P = 0.364 ; n.s.	$F_{(1,30)}$ = 6.29; P = 0.018 ; *
50-150 Hz	$F_{(1,30)}$ = 17.30; P < 0.001 ; ***	$F_{(1,30)}$ = 11.82; P = 0.002 ; **	$F_{(1,30)}$ = 27.21; P < 0.001 ; ***
**CA1**	**WT vs. PVKO: LFP spectral powers measured from the hippocampal CA1 area (CA1)**		
**Band**	**Fig. 2A: Going to the lever**	**Fig. 2B: Pressing the lever**	**Fig. 2C: Going to the feeder**
1-4 Hz	$F_{(1,30)}$ = 21.16; P < 0.001 ; ***	$F_{(1,30)}$ = 11.43; P = 0.002 ; **	$F_{(1,30)}$ = 26.21; P < 0.001 ; ***
4-12 Hz	$F_{(1,30)}$ = 25.27; P < 0.001 ; ***	$F_{(1,30)}$ = 18.25; P < 0.001 ; ***	$F_{(1,30)}$ = 22.06; P < 0.001 ; ***
12-30 Hz	$F_{(1,30)}$ = 38.43; P < 0.001 ; ***	$F_{(1,30)}$ = 16.75; P < 0.001 ; ***	$F_{(1,30)}$ = 17.91; P < 0.001 ; ***
30-50 Hz	$F_{(1,30)}$ = 19.05; P < 0.001 ; ***	$F_{(1,30)}$ = 17.38; P < 0.001 ; ***	$F_{(1,30)}$ = 17.59; P < 0.001 ; ***
50-150 Hz	$F_{(1,30)}$ = 9.65; P = 0.004 ; **	$F_{(1,30)}$ = 12.16; P = 0.002 ; **	$F_{(1,30)}$ = 11.85; P = 0.002 ; **
**MS**	**WT vs. PVKO: LFP spectral powers measured from the medial septum (MS)**		
**Band**	**Fig. 2A: Going to the lever**	**Fig. 2B: Pressing the lever**	**Fig. 2C: Going to the feeder**
1-4 Hz	$F_{(1,30)}$ = 3.83; P = 0.060 ; n.s.	$F_{(1,30)}$ = 5.71; P = 0.023 ; *	$F_{(1,30)}$ = 3.78; P = 0.061 ; n.s.
4-12 Hz	$F_{(1,30)}$ = 5.06; P = 0.032 ; *	$F_{(1,30)}$ = 10.35; P = 0.003 ; **	$F_{(1,30)}$ = 2.34; P = 0.137 ; n.s.
12-30 Hz	$F_{(1,30)}$ = 5.49; P = 0.026 ; *	$F_{(1,30)}$ = 0.25; P = 0.620 ; n.s.	$F_{(1,30)}$ = 0.03; P = 0.924 ; n.s.
30-50 Hz	$F_{(1,30)}$ = 0.01; P = 0.934 ; n.s.	$F_{(1,30)}$ = 6.20; P = 0.019 ; *	$F_{(1,30)}$ = 0.67; P = 0.421 ; n.s.
50-150 Hz	$F_{(1,30)}$ = 1.38; P = 0.250 ; n.s.	$F_{(1,30)}$ = 8.19; P = 0.008 ; **	$F_{(1,30)}$ = 0.89; P = 0.352 ; n.s.
**MD**	**WT vs. PVKO: LFP spectral powers measured from the mediodorsal thalamic nucleus (MD)**		
**Band**	**Fig. 2A: Going to the lever**	**Fig. 2B: Pressing the lever**	**Fig. 2C: Going to the feeder**
1-4 Hz	$F_{(1,30)}$ = 10.35; P = 0.003 ; **	$F_{(1,30)}$ = 7.20; P = 0.012 ; *	$F_{(1,30)}$ = 7.48; P = 0.010 ; *
4-12 Hz	$F_{(1,30)}$ = 4.24; P = 0.048 ; *	$F_{(1,30)}$ = 2.63; P = 0.115 ; n.s.	$F_{(1,30)}$ = 0.43; P = 0.519 ; n.s.
12-30 Hz	$F_{(1,30)}$ = 0.33; P = 0.570 ; n.s.	$F_{(1,30)}$ = 18.33; P < 0.001 ; ***	$F_{(1,30)}$ = 8.21; P = 0.008 ; **
30-50 Hz	$F_{(1,30)}$ = 0.13; P = 0.723 ; n.s.	$F_{(1,30)}$ = 14.27; P < 0.001 ; ***	$F_{(1,30)}$ = 2.92; P = 0.098 ; n.s.
50-150 Hz	$F_{(1,30)}$ = 1.00; P = 0.325 ; n.s.	$F_{(1,30)}$ = 6.95; P = 0.013 ; *	$F_{(1,30)}$ = 2.88; P = 0.100 ; n.s.

Note that : n.s. indicates non-significant differences; *, P < 0.05; **, P < 0.01; ***, P < 0.001.
Yellow boxes highlight comparisos characterized by rounded $F_{(1,30)}$ value larger than 10.

Table S3. Statistical reports corresponding to the multiple comparisons for the probability density measured **from the five selected sites** and between the defined pairs of experimental conditions (see **Fig. 3** in the main text).

**mPFC: medial prefrontal cortex [Fig. 3B]**		**Inference Type and Probability Density (Mean ± SEM)**		
**Probabilistic Map at the right of the panel**	**Criterium**	**Type –1 (blue)**	**Type 0 (green)**	**Type + 1 (brown)**
PVKO (correct) vs. PVKO (incorrect)	NO	17 ± 1	57 ± 1	27 ± 1
PVKO (correct) vs. WT (correct)	**YES**	4 ± 1	12 ± 1	84 ± 1
PVKO (incorrect) vs. WT (incorrect)	NO	15 ± 1	53 ± 1	33 ± 1
WT (correct) vs. WT (incorrect)	**YES**	75 ± 1	19 ± 1	6 ± 1
**NAc: nucleus accumbens [Fig. 3C]**		**Inference Type and Probability Density (Mean ± SEM)**		
**Probabilistic Map at the right of the panel**	**Criterium**	**Type –1 (blue)**	**Type 0 (green)**	**Type + 1 (brown)**
PVKO (correct) vs. PVKO (incorrect)	NO	28 ± 1	55 ± 1	17 ± 1
PVKO (correct) vs. WT (correct)	**YES**	16 ± 1	24 ± 1	60 ± 1
PVKO (incorrect) vs. WT (incorrect)	NO	33 ± 1	51 ± 1	17 ± 1
WT (correct) vs. WT (incorrect)	**YES**	71 ± 1	26 ± 1	3 ± 1
**CA1: hippocampal CA1 area [Fig. 3D]**		**Inference Type and Probability Density (Mean ± SEM)**		
**Probabilistic Map at the right of the panel**	**Criterium**	**Type –1 (blue)**	**Type 0 (green)**	**Type + 1 (brown)**
PVKO (correct) vs. PVKO (incorrect)	NO	23 ± 1	46 ± 1	30 ± 1
PVKO (correct) vs. WT (correct)	NO	32 ± 1	34 ± 1	34 ± 1
PVKO (incorrect) vs. WT (incorrect)	**YES**	74 ± 1	25 ± 1	2 ± 1
WT (correct) vs. WT (incorrect)	**YES**	68 ± 1	30 ± 1	3 ± 1
**MS: medial septum [n.r.]**		**Inference Type and Probability Density (Mean ± SEM)**		
**Probabilistic Map at the right of the panel**	**Criterium**	**Type –1 (blue)**	**Type 0 (green)**	**Type + 1 (brown)**
PVKO (correct) vs. PVKO (incorrect)	NO	33 ± 1	58 ± 1	9 ± 1
PVKO (correct) vs. WT (correct)	NO	8 ± 1	54 ± 1	38 ± 1
PVKO (incorrect) vs. WT (incorrect)	NO	10 ± 1	43 ± 1	47 ± 1
WT (correct) vs. WT (incorrect)	NO	36 ± 1	44 ± 1	20 ± 1
**MD: mediodorsal thalamic nucleus [n.r.]**		**Inference Type and Probability Density (Mean ± SEM)**		
**Probabilistic Map at the right of the panel**	**Criterium**	**Type –1 (blue)**	**Type 0 (green)**	**Type + 1 (brown)**
PVKO (correct) vs. PVKO (incorrect)	**YES**	61 ± 1	24 ± 1	15 ± 1
PVKO (correct) vs. WT (correct)	NO	6 ± 1	49 ± 1	46 ± 1
PVKO (incorrect) vs. WT (incorrect)	NO	9 ± 1	36 ± 1	55 ± 1
WT (correct) vs. WT (incorrect)	NO	49 ± 1	36 ± 1	15 ± 1

Here n.r. indicates result not represented in the main text (for MS and MD sites). The selected statistical criterium was that condition 1 (inference type ±1 representing > 56.7% of the probability density) and condition 2 (inference type 0 representing < 33.3% of the probability density) were simultaneously verified. Inference type –1, E_{1st} (the estimate of power in the first spectrogram) $\gg$ E_{2nd} (the estimate of power in the second spectrogram). Inference type +1, E_{2nd} (the estimate of power in the second spectrogram) $\gg$ E_{1st} (the estimate of power in the first spectrogram). Inference type 0, E_{1st} (the estimate of power in the first spectrogram) $\approx$ E_{2nd} (the estimate of power in the second spectrogram). SEM: standard error of the mean.

Table S4.1. Statistical reports corresponding to the multiple comparisons for the mean values of the LFP spectral powers measured **from the mPFC** (see **Fig. 4B,C**) respect to the **baseline condition**.

**Fig. 4B: LFPs recorded from Beginning (two first min.) and End (two last min.) of Sessions 4 to 8.**				
**Band 1-4 Hz: Pairwise comparison**	**Confidence Interval (95%)**			***P*-value**
WT-Beginning vs. WT-Baseline	19.498	39.597	59.696	< 0.001 ***
WT-End vs. WT-Baseline	17.994	38.093	58.193	< 0.001 ***
PVKO-Beginning vs. PVKO-Baseline	20.031	40.130	60.230	< 0.001 ***
PVKO-End vs. PVKO-Baseline	15.051	35.150	55.250	< 0.001 ***
WT-Baseline vs. PVKO-Baseline	1.889	21.988	42.088	0.024 *
**Band 4-12 Hz: Pairwise comparison**	**Confidence Interval (95%)**			***P*-value**
WT-Beginning vs. WT-Baseline	11.634	37.944	64.254	< 0.001 ***
WT-End vs. WT-Baseline	23.229	49.539	75.849	< 0.001 ***
PVKO-Beginning vs. PVKO-Baseline	−1.063	25.247	51.558	0.067 n.s.
PVKO-End vs. PVKO-Baseline	−4.193	22.117	48.427	0.149 n.s.
WT-Baseline vs. PVKO-Baseline	−12.939	13.372	39.682	0.671 n.s.
**Band 12-30 Hz: Pairwise comparison**	**Confidence Interval (95%)**			***P*-value**
WT-Beginning vs. WT-Baseline	7.419	14.620	21.820	< 0.001 ***
WT-End vs. WT-Baseline	8.657	15.858	23.058	< 0.001 ***
PVKO-Beginning vs. PVKO-Baseline	−5.071	2.129	9.330	0.953 n.s.
PVKO-End vs. PVKO-Baseline	−5.961	1.240	8.440	0.996 n.s.
WT-Baseline vs. PVKO-Baseline	−8.422	−1.221	5.979	0.996 n.s.
**Band 30-50 Hz: Pairwise comparison**	**Confidence Interval (95%)**			***P*-value**
WT-Beginning vs. WT-Baseline	11.590	14.133	16.676	< 0.001 ***
WT-End vs. WT-Baseline	10.435	12.978	15.521	< 0.001 ***
PVKO-Beginning vs. PVKO-Baseline	−5.341	−2.799	−0.256	0.023 *
PVKO-End vs. PVKO-Baseline	−5.863	−3.320	−0.777	0.004 **
WT-Baseline vs. PVKO-Baseline	−7.398	−4.855	−2.312	< 0.001 ***
**Band 50-150 Hz: Pairwise comparison**	**Confidence Interval (95%)**			***P*-value**
WT-Beginning vs. WT-Baseline	4.149	5.026	5.903	< 0.001 ***
WT-End vs. WT-Baseline	4.728	5.605	6.481	< 0.001 ***
PVKO-Beginning vs. PVKO-Baseline	−0.767	0.110	0.987	0.999 n.s.
PVKO-End vs. PVKO-Baseline	−0.964	−0.087	0.789	0.999 n.s.
WT-Baseline vs. PVKO-Baseline	−1.273	−0.396	0.481	0.770 n.s.
**Fig. 4B: LFPs recorded from operant conditioning Session 1 vs. Session 10.**				
**Band 1-4 Hz: Pairwise comparison**	**Confidence Interval (95%)**			***P*-value**
WT-Session 1 vs. WT-Baseline	37.862	64.204	90.546	< 0.001 ***
WT-Session 10 vs. WT-Baseline	0.038	26.380	52.722	0.049 *
PVKO-Session 1 vs. PVKO-Baseline	8.494	34.836	61.178	0.003 **
PVKO-Session 10 vs. PVKO-Baseline	−4.262	22.078	48.422	0.151 n.s.
WT-Baseline vs. PVKO-Baseline	−4.354	21.988	48.331	0.155 n.s.
**Band 4-12 Hz: Pairwise comparison**	**Confidence Interval (95%)**			***P*-value**
WT-Session 1 vs. WT-Baseline	12.627	32.596	52.565	< 0.001 ***
WT-Session 10 vs. WT-Baseline	25.607	45.576	65.545	< 0.001 ***
PVKO-Session 1 vs. PVKO-Baseline	9.830	29.799	49.768	< 0.001 ***
PVKO-Session 10 vs. PVKO-Baseline	−3.406	16.563	36.532	0.160 n.s.
WT-Baseline vs. PVKO-Baseline	−6.597	13.372	33.341	0.373 n.s
**Band 12-30 Hz: Pairwise comparison**	**Confidence Interval (95%)**			***P*-value**
WT-Session 1 vs. WT-Baseline	7.684	14.231	20.777	< 0.001 ***
WT-Session 10 vs. WT-Baseline	7.064	13.611	20.157	< 0.001 ***
PVKO-Session 1 vs. PVKO-Baseline	−5.065	1.482	8.028	0.985 n.s.
PVKO-Session 10 vs. PVKO-Baseline	−4.568	1.978	8.524	0.948 n.s.
WT-Baseline vs. PVKO-Baseline	−7.767	−1.221	5.325	0.994 n.s.
**Band 30-50 Hz: Pairwise comparison**	**Confidence Interval (95%)**			***P*-value**
WT-Session 1 vs. WT-Baseline	12.298	16.902	21.506	< 0.001 ***
WT-Session 10 vs. WT-Baseline	8.774	13.378	17.982	< 0.001 ***
PVKO-Session 1 vs. PVKO-Baseline	−2.966	1.638	6.242	0.901 n.s.
PVKO-Session 10 vs. PVKO-Baseline	−5.226	−0.622	3.982	0.999 n.s.
WT-Baseline vs. PVKO-Baseline	−9.459	−4.855	−0.251	0.033 *
**Band 50-150 Hz: Pairwise comparison**	**Confidence Interval (95%)**			***P*-value**
WT-Session 1 vs. WT-Baseline	3.867	5.497	7.127	< 0.001 ***
WT-Session 10 vs. WT-Baseline	4.060	5.690	7.320	< 0.001 ***
PVKO-Session 1 vs. PVKO-Baseline	0.944	2.574	4.204	< 0.001 ***
PVKO-Session 10 vs. PVKO-Baseline	−0.302	1.328	2.958	0.175 n.s.
WT-Baseline vs. PVKO-Baseline	−2.026	−0.396	1.234	0.980 n.s

Note that: n.s. indicates non-significant differences; *, $P < 0.05$; **, $P < 0.01$; ***, $P < 0.001$.

Table S4.2. Statistical reports corresponding to the multiple comparisons for the mean values of the LFP spectral powers measured **from the mPFC** (see **Fig. 4B** in the main text).

**Fig. 4B: LFPs recorded from the Beginning (two first min.) and the End (two last min.) of Sessions 4 to 8.**				
**Band 1-4 Hz: Many Groups**	**Statistical Tests and Reports**			***P*-value**
WT-Beginning vs. WT-End vs. PVKO-Beginning vs. PVKO-End	One-Way ANOVA *F*-test: $F_{(3,44)} = 6.60$ Tukey-Kramer test for the multiple comparisons			< 0.001 ***
**Band 1-4 Hz: Pairwise comparison**	**Confidence Interval (95%)**			
WT-Beginning vs. WT-End	−18.424	1.504	21.431	0.997 n.s.
WT-Beginning vs. PVKO-Beginning	1.527	21.455	41.383	0.030 *
WT-Beginning vs. PVKO-End	6.507	26.435	46.362	0.005 **
WT-End vs. PVKO-Beginning	0.024	19.951	39.879	0.050 *
WT-End vs. PVKO-End	5.003	24.931	44.858	0.009 **
PVKO-Beginning vs. PVKO-End	−14.948	4.980	24.907	0.910 n.s.
**Band 4-12 Hz: Many Groups**	**Statistical Tests and Reports**			***P*-value**
WT-Beginning vs. WT-End vs. PVKO-Beginning vs. PVKO-End	One-Way ANOVA *F*-test: $F_{(3,44)} = 7.11$ Tukey-Kramer test for the multiple comparisons			< 0.001 ***
**Band 4-12 Hz: Pairwise comparison**	**Confidence Interval (95%)**			
WT-Beginning vs. WT-End	−39.787	−11.594	16.598	0.693 n.s.
WT-Beginning vs. PVKO-Beginning	−2.124	26.069	54.261	0.791 n.s.
WT-Beginning vs. PVKO-End	1.007	29.199	57.391	0.040 *
WT-End vs. PVKO-Beginning	9.471	37.663	65.855	0.005 **
WT-End vs. PVKO-End	12.601	40.793	68.985	0.002 **
PVKO-Beginning vs. PVKO-End	−25.062	3.130	31.322	0.991 n.s.
**Band 12-30 Hz: Many Groups**	**Statistical Tests and Reports**			***P*-value**
WT-Beginning vs. WT-End vs. PVKO-Beginning vs. PVKO-End	One-Way ANOVA *F*-test: $F_{(3,44)} = 12.46$ Tukey-Kramer test for the multiple comparisons			< 0.001 ***
**Band 12-30 Hz: Pairwise comparison**	**Confidence Interval (95%)**			
WT-Beginning vs. WT-End	−8.884	−1.238	6.408	0.973 n.s.
WT-Beginning vs. PVKO-Beginning	3.623	11.269	18.915	0.002 **
WT-Beginning vs. PVKO-End	4.513	12.159	19.805	< 0.001 ***
WT-End vs. PVKO-Beginning	4.861	12.507	20.153	< 0.001 ***
WT-End vs. PVKO-End	5.751	13.397	21.043	< 0.001 ***
PVKO-Beginning vs. PVKO-End	−6.756	0.890	8.536	0.989 n.s.
**Band 30-50 Hz: Many Groups)**	**Statistical Tests and Reports**			***P*-value**
WT-Beginning vs. WT-End vs. PVKO-Beginning vs. PVKO-End	One-Way ANOVA *F*-test: $F_{(3,44)} = 119.98$ Tukey-Kramer test for the multiple comparisons			< 0.001 ***
**Band 30-50 Hz: Pairwise comparison**	**Confidence Interval (95%)**			
WT-Beginning vs. WT-End	−1.193	1.155	3.502	0.560 n.s.
WT-Beginning vs. PVKO-Beginning	9.729	12.076	14.423	< 0.001 ***
WT-Beginning vs. PVKO-End	10.250	12.597	14.945	< 0.001 ***
WT-End vs. PVKO-Beginning	8.574	10.922	13.269	< 0.001 ***
WT-End vs. PVKO-End	9.096	11.443	13.790	< 0.001 ***
PVKO-Beginning vs. PVKO-End	−1.826	0.521	2.869	0.934 n.s.
**Band 50-150 Hz: Many Groups**	**Statistical Tests and Reports**			***P*-value**
WT-Beginning vs. WT-End vs. PVKO-Beginning vs. PVKO-End	One-Way ANOVA *F*-test: $F_{(3,44)} = 147.93$ Tukey-Kramer test for the multiple comparisons			< 0.001 ***
**Band 50-150 Hz: Pairwise comparison**	**Confidence Interval (95%)**			
WT-Beginning vs. WT-End	−1.462	−0.579	0.304	0.311 n.s.
WT-Beginning vs. PVKO-Beginning	3.637	4.520	5.403	< 0.001 ***
WT-Beginning vs. PVKO-End	3.834	4.717	5.600	< 0.001 ***
WT-End vs. PVKO-Beginning	4.216	5.099	5.982	< 0.001 ***
WT-End vs. PVKO-End	4.413	5.296	6.179	< 0.001 ***
PVKO-Beginning vs. PVKO-End	−0.686	0.197	1.081	0.933 n.s.

Note that: n.s. indicates non-significant differences; *, $P < 0.05$; **, $P < 0.01$; ***, $P < 0.001$.

Table S4.3. Statistical reports corresponding to the multiple comparisons for the mean values of the LFP spectral powers measured **from the mPFC** (see **Fig. 4C** in the main text).

**Fig. 4B: LFPs recorded from operant conditioning Session 1 vs. Session 10.**				
**Band 1-4 Hz: Many Groups**	**Statistical Tests and Reports**			***P*-value**
WT-Session 1 vs. WT-Session 10 vs. PVKO-Session 1 vs. PVKO-Session 10	One-Way ANOVA *F*-test: $F_{(3,44)}$ = 14.46<br>Tukey-Kramer test for the multiple comparisons			< 0.001 ***
**Band 1-4 Hz: Pairwise comparison**	**Confidence Interval (95%)**			
WT-Session 1 vs. WT-Session 10	10.310	37.824	65.337	0.004 **
WT-Session 1 vs. PVKO-Session 1	23.843	51.357	78.870	< 0.001 ***
WT-Session 1 vs. PVKO-Session 10	36.599	64.113	91.626	< 0.001 ***
WT-Session 10 vs. PVKO-Session 1	−13.981	13.533	41.046	0.560 n.s.
WT-Session 10 vs. PVKO-Session 10	−1.225	26.289	53.802	0.066 n.s.
PVKO-Session 1 vs. PVKO-Session 10	−14.758	12.756	40.269	0.607 n.s.
**Band 4-12 Hz: Many Groups**	**Statistical Tests and Reports**			***P*-value**
WT-Session 1 vs. WT-Session 10 vs. PVKO-Session 1 vs. PVKO-Session 10	One-Way ANOVA *F*-test: $F_{(3,44)}$ = 11.36<br>Tukey-Kramer test for the multiple comparisons			< 0.001 ***
**Band 4-12 Hz: Pairwise comparison**	**Confidence Interval (95%)**			
WT-Session 1 vs. WT-Session 10	−33.730	−12.981	7.769	0.351 n.s.
WT-Session 1 vs. PVKO-Session 1	−4.581	16.168	36.918	0.175 n.s.
WT-Session 1 vs. PVKO-Session 10	8.655	29.404	50.154	0.003 **
WT-Session 10 vs. PVKO-Session 1	8.400	29.149	49.898	0.003 **
WT-Session 10 vs. PVKO-Session 10	21.635	42.385	63.134	< 0.001 ***
PVKO-Session 1 vs. PVKO-Session 10	−7.514	13.236	33.985	0.334 n.s.
**Band 12-30 Hz: Many Groups**	**Statistical Tests and Reports**			***P*-value**
WT-Session 1 vs. WT-Session 10 vs. PVKO-Session 1 vs. PVKO-Session 10	One-Way ANOVA *F*-test: $F_{(3,44)}$ = 12.12<br>Tukey-Kramer test for the multiple comparisons			< 0.001 ***
**Band 12-30 Hz: Pairwise comparison**	**Confidence Interval (95%)**			
WT-Session 1 vs. WT-Session 10	−6.257	0.620	7.497	0.995 n.s.
WT-Session 1 vs. PVKO-Session 1	4.651	11.528	18.405	< 0.001 ***
WT-Session 1 vs. PVKO-Session 10	4.154	11.031	17.909	< 0.001 ***
WT-Session 10 vs. PVKO-Session 1	4.031	10.908	17.785	< 0.001 ***
WT-Session 10 vs. PVKO-Session 10	3.534	10.411	17.289	0.001 **
PVKO-Session 1 vs. PVKO-Session 10	−7.374	−0.497	6.381	0.997 n.s.
**Band 30-50 Hz: Many Groups)**	**Statistical Tests and Reports**			***P*-value**
WT-Session 1 vs. WT-Session 10 vs. PVKO-Session 1 vs. PVKO-Session 10	One-Way ANOVA *F*-test: $F_{(3,44)}$ = 20.84<br>Tukey-Kramer test for the multiple comparisons			< 0.001 ***
**Band 30-50 Hz: Pairwise comparison**	**Confidence Interval (95%)**			
WT-Session 1 vs. WT-Session 10	−1.354	3.524	8.402	0.231 n.s.
WT-Session 1 vs. PVKO-Session 1	5.530	10.408	15.286	< 0.001 ***
WT-Session 1 vs. PVKO-Session 10	7.791	12.669	17.547	< 0.001 ***
WT-Session 10 vs. PVKO-Session 1	2.006	6.884	11.762	0.003 **
WT-Session 10 vs. PVKO-Session 10	4.267	9.145	14.023	< 0.001 ***
PVKO-Session 1 vs. PVKO-Session 10	−2.618	2.260	7.138	0.607 n.s.
**Band 50-150 Hz: Many Groups**	**Statistical Tests and Reports**			***P*-value**
WT-Session 1 vs. WT-Session 10 vs. PVKO-Session 1 vs. PVKO-Session 10	One-Way ANOVA *F*-test: $F_{(3,44)}$ = 17.25<br>Tukey-Kramer test for the multiple comparisons			< 0.001 ***
**Band 50-150 Hz: Pairwise comparison**	**Confidence Interval (95%)**			
WT-Session 1 vs. WT-Session 10	−1.961	−0.193	1.574	0.991 n.s.
WT-Session 1 vs. PVKO-Session 1	0.760	2.527	4.294	0.002 **
WT-Session 1 vs. PVKO-Session 10	2.006	3.773	5.540	< 0.001 ***
WT-Session 10 vs. PVKO-Session 1	0.953	2.720	4.488	< 0.001 ***
WT-Session 10 vs. PVKO-Session 10	2.199	3.966	5.734	< 0.001 ***
PVKO-Session 1 vs. PVKO-Session 10	−0.521	1.246	3.013	0.250 n.s.

Note that: n.s. indicates non-significant differences; *, $P < 0.05$; **, $P < 0.01$; ***, $P < 0.001$.

Table S5.1. Statistical reports corresponding to the multiple comparisons for the mean values of the LFP spectral powers measured **from the NAc** (see **Fig. 5B,C**) respect to the **baseline condition**.

**Fig. 5B: LFPs recorded from Beginning (two first min.) and End (two last min.) of Sessions 4 to 8.**				
**Band 1-4 Hz: Pairwise comparison**	**Confidence Interval (95%)**			***P*-value**
WT-Beginning vs. WT-Baseline	49.844	75.653	101.460	< 0.001 ***
WT-End vs. WT-Baseline	44.961	70.770	96.579	< 0.001 ***
PVKO-Beginning vs. PVKO-Baseline	0.918	26.727	52.536	0.038 *
PVKO-End vs. PVKO-Baseline	−0.820	24.989	50.798	0.063 n.s.
WT-Baseline vs. PVKO-Baseline	−20.840	4.968	30.777	0.993 n.s.
**Band 4-12 Hz: Pairwise comparison**	**Confidence Interval (95%)**			***P*-value**
WT-Beginning vs. WT-Baseline	47.816	74.730	101.640	< 0.001 ***
WT-End vs. WT-Baseline	61.731	88.645	115.560	< 0.001 ***
PVKO-Beginning vs. PVKO-Baseline	−6.753	20.161	47.074	0.252 n.s.
PVKO-End vs. PVKO-Baseline	−13.139	13.775	40.689	0.664 n.s.
WT-Baseline vs. PVKO-Baseline	−33.041	−6.127	20.786	0.985 n.s.
**Band 12-30 Hz: Pairwise comparison**	**Confidence Interval (95%)**			***P*-value**
WT-Beginning vs. WT-Baseline	24.640	30.783	36.925	< 0.001 ***
WT-End vs. WT-Baseline	23.432	29.575	35.717	< 0.001 ***
PVKO-Beginning vs. PVKO-Baseline	5.274	11.416	17.558	< 0.001 ***
PVKO-End vs. PVKO-Baseline	4.149	10.291	16.434	< 0.001 ***
WT-Baseline vs. PVKO-Baseline	−1.893	4.249	10.392	0.337 n.s.
**Band 30-50 Hz: Pairwise comparison**	**Confidence Interval (95%)**			***P*-value**
WT-Beginning vs. WT-Baseline	19.010	24.179	29.349	< 0.001 ***
WT-End vs. WT-Baseline	17.722	22.891	28.061	< 0.001 ***
PVKO-Beginning vs. PVKO-Baseline	−2.276	2.893	8.063	0.574 n.s.
PVKO-End vs. PVKO-Baseline	−2.943	2.227	7.396	0.803 n.s.
WT-Baseline vs. PVKO-Baseline	−5.770	−0.600	4.569	0.999 n.s.
**Band 50-150 Hz: Pairwise comparison**	**Confidence Interval (95%)**			***P*-value**
WT-Beginning vs. WT-Baseline	5.750	6.699	7.647	< 0.001 ***
WT-End vs. WT-Baseline	6.707	7.656	8.605	< 0.001 ***
PVKO-Beginning vs. PVKO-Baseline	−1.840	−0.891	0.058	0.776 n.s.
PVKO-End vs. PVKO-Baseline	−2.138	−1.189	−0.240	0.006 **
WT-Baseline vs. PVKO-Baseline	−1.312	−0.364	0.585	0.870 n.s.

**Fig. 5B: LFPs recorded from operant conditioning Session 1 vs. Session 10.**				
**Band 1-4 Hz: Pairwise comparison**	**Confidence Interval (95%)**			***P*-value**
WT-Session 1 vs. WT-Baseline	−12.735	10.602	33.939	0.765 n.s.
WT-Session 10 vs. WT-Baseline	30.621	53.958	77.295	< 0.001 ***
PVKO-Session 1 vs. PVKO-Baseline	19.411	42.748	66.084	< 0.001 ***
PVKO-Session 10 vs. PVKO-Baseline	−10.564	12.773	36.110	0.597 n.s.
WT-Baseline vs. PVKO-Baseline	18.369	4.968	28.305	0.989 n.s.
**Band 4-12 Hz: Pairwise comparison**	**Confidence Interval (95%)**			***P*-value**
WT-Session 1 vs. WT-Baseline	−5.070	18.314	41.698	0.209 n.s.
WT-Session 10 vs. WT-Baseline	60.726	84.100	107.490	< 0.001 ***
PVKO-Session 1 vs. PVKO-Baseline	24.680	48.064	71.448	< 0.001 ***
PVKO-Session 10 vs. PVKO-Baseline	−8.853	14.531	37.915	0.458 n.s
WT-Baseline vs. PVKO-Baseline	29.511	−6.127	17.257	0.972 n.s.
**Band 12-30 Hz: Pairwise comparison**	**Confidence Interval (95%)**			***P*-value**
WT-Session 1 vs. WT-Baseline	−2.816	5.618	14.051	0.379 n.s.
WT-Session 10 vs. WT-Baseline	18.034	26.468	34.901	< 0.001 ***
PVKO-Session 1 vs. PVKO-Baseline	14.712	23.145	31.578	< 0.001 ***
PVKO-Session 10 vs. PVKO-Baseline	4.890	13.323	21.757	< 0.001 ***
WT-Baseline vs. PVKO-Baseline	−4.184	4.249	12.683	0.679 n.s.
**Band 30-50 Hz: Pairwise comparison**	**Confidence Interval (95%)**			***P*-value**
WT-Session 1 vs. WT-Baseline	6.714	12.816	18.918	< 0.001 ***
WT-Session 10 vs. WT-Baseline	15.721	21.822	27.924	< 0.001 ***
PVKO-Session 1 vs. PVKO-Baseline	16.212	22.313	28.415	< 0.001 ***
PVKO-Session 10 vs. PVKO-Baseline	−0.404	5.698	11.799	0.807 n.s.
WT-Baseline vs. PVKO-Baseline	−6.702	−0.600	5.501	0.999 n.s.
**Band 50-150 Hz: Pairwise comparison**	**Confidence Interval (95%)**			***P*-value**
WT-Session 1 vs. WT-Baseline	3.117	4.428	5.739	< 0.001 ***
WT-Session 10 vs. WT-Baseline	6.483	7.794	9.105	< 0.001 ***
PVKO-Session 1 vs. PVKO-Baseline	7.064	8.376	9.687	< 0.001 ***
PVKO-Session 10 vs. PVKO-Baseline	−0.934	0.377	1.688	0.958 n.s.
WT-Baseline vs. PVKO-Baseline	−1.675	−0.364	0.948	0.964 n.s.

Note that: n.s. indicates non-significant differences; *, $P < 0.05$; **, $P < 0.01$; ***, $P < 0.001$.

Table S5.2. Statistical reports corresponding to the multiple comparisons for the mean values of the LFP spectral powers measured **from the NAc** (see **Fig. 5B** in the main text).

**Fig. 5B: LFPs recorded from the Beginning (two first min.) and the End (two last min.) of Sessions 4 to 8.**				
**Band 1-4 Hz: Many Groups**	**Statistical Tests and Reports**			***P*-value**
WT-Beginning vs. WT-End vs. PVKO-Beginning vs. PVKO-End	One-Way ANOVA *F*-test: $F_{(3,44)} = 17.41$<br>Tukey-Kramer test for the multiple comparisons			< 0.001 ***
**Band 1-4 Hz: Pairwise comparison**	**Confidence Interval (95%)**			
WT-Beginning vs. WT-End	−22.522	4.883	32.287	0.964 n.s.
WT-Beginning vs. PVKO-Beginning	26.489	53.894	81.299	< 0.001 ***
WT-Beginning vs. PVKO-End	28.227	55.632	83.037	< 0.001 ***
WT-End vs. PVKO-Beginning	21.607	49.011	76.416	< 0.001 ***
WT-End vs. PVKO-End	23.345	50.749	78.154	< 0.001 ***
PVKO-Beginning vs. PVKO-End	−25.667	1.738	29.143	0.998 n.s.
**Band 4-12 Hz: Many Groups**	**Statistical Tests and Reports**			***P*-value**
WT-Beginning vs. WT-End vs. PVKO-Beginning vs. PVKO-End	One-Way ANOVA *F*-test: $F_{(3,44)} = 19.62$<br>Tukey-Kramer test for the multiple comparisons			< 0.001 ***
**Band 4-12 Hz: Pairwise comparison**	**Confidence Interval (95%)**			
WT-Beginning vs. WT-End	−43.240	−13.915	15.410	0.588 n.s.
WT-Beginning vs. PVKO-Beginning	19.116	48.442	77.767	< 0.001 ***
WT-Beginning vs. PVKO-End	25.502	54.827	84.152	< 0.001 ***
WT-End vs. PVKO-Beginning	33.031	62.357	91.682	< 0.001 ***
WT-End vs. PVKO-End	39.417	68.742	98.067	< 0.001 ***
PVKO-Beginning vs. PVKO-End	−22.940	6.386	35.711	0.937 n.s.
**Band 12-30 Hz: Many Groups**	**Statistical Tests and Reports**			***P*-value**
WT-Beginning vs. WT-End vs. PVKO-Beginning vs. PVKO-End	One-Way ANOVA *F*-test: $F_{(3,44)} = 59.86$<br>Tukey-Kramer test for the multiple comparisons			< 0.001 ***
**Band 12-30 Hz: Pairwise comparison**	**Confidence Interval (95%)**			
WT-Beginning vs. WT-End	−5.443	1.208	7.859	0.962 n.s.
WT-Beginning vs. PVKO-Beginning	16.965	23.616	30.267	< 0.001 ***
WT-Beginning vs. PVKO-End	18.090	24.741	31.391	< 0.001 ***
WT-End vs. PVKO-Beginning	15.757	22.408	29.059	< 0.001 ***
WT-End vs. PVKO-End	16.882	23.533	30.183	< 0.001 ***
PVKO-Beginning vs. PVKO-End	−5.526	1.125	7.775	0.969 n.s.
**Band 30-50 Hz: Many Groups)**	**Statistical Tests and Reports**			***P*-value**
WT-Beginning vs. WT-End vs. PVKO-Beginning vs. PVKO-End	One-Way ANOVA *F*-test: $F_{(3,44)} = 98.35$<br>Tukey-Kramer test for the multiple comparisons			< 0.001 ***
**Band 30-50 Hz: Pairwise comparison**	**Confidence Interval (95%)**			
WT-Beginning vs. WT-End	−3.197	1.288	5.773	0.869 n.s.
WT-Beginning vs. PVKO-Beginning	16.201	20.686	25.170	< 0.001 ***
WT-Beginning vs. PVKO-End	16.868	21.352	25.837	< 0.001 ***
WT-End vs. PVKO-Beginning	14.913	19.397	23.882	< 0.001 ***
WT-End vs. PVKO-End	15.579	20.064	24.549	< 0.001 ***
PVKO-Beginning vs. PVKO-End	−3.818	0.667	5.151	0.979 n.s.
**Band 50-150 Hz: Many Groups**	**Statistical Tests and Reports**			***P*-value**
WT-Beginning vs. WT-End vs. PVKO-Beginning vs. PVKO-End	One-Way ANOVA *F*-test: $F_{(3,44)} = 326.19$<br>Tukey-Kramer test for the multiple comparisons			< 0.001 ***
**Band 50-150 Hz: Pairwise comparison**	**Confidence Interval (95%)**			
WT-Beginning vs. WT-End	−1.909	−0.957	−0.006	0.048 *
WT-Beginning vs. PVKO-Beginning	6.275	7.226	8.178	< 0.001 ***
WT-Beginning vs. PVKO-End	6.572	7.524	8.476	< 0.001 ***
WT-End vs. PVKO-Beginning	7.232	8.184	9.136	< 0.001 ***
WT-End vs. PVKO-End	7.529	8.481	9.433	< 0.001 ***
PVKO-Beginning vs. PVKO-End	−0.655	0.297	1.249	0.838 n.s.

Note that: n.s. indicates non-significant differences; *, $P < 0.05$; **, $P < 0.01$; ***, $P < 0.001$.

Table S5.3. Statistical reports corresponding to the multiple comparisons for the mean values of the LFP spectral powers measured **from the NAc** (see **Fig. 5C** in the main text).

**Fig. 5B: LFPs recorded from operant conditioning Session 1 vs. Session 10.**				
**Band 1-4 Hz: Many Groups**	**Statistical Tests and Reports**			***P*-value**
WT-Session 1 vs. WT-Session 10 vs. PVKO-Session 1 vs. PVKO-Session 10	One-Way ANOVA *F*-test: $F_{(3,44)}$ = 11.71<br>Tukey-Kramer test for the multiple comparisons			< 0.001 ***
**Band 1-4 Hz: Pairwise comparison**	**Confidence Interval (95%)**			
WT-Session 1 vs. WT-Session 10	−67.855	−43.356	−18.856	< 0.001 ***
WT-Session 1 vs. PVKO-Session 1	−51.677	−27.177	−2.678	0.024 *
WT-Session 1 vs. PVKO-Session 10	−21.703	2.797	27.296	0.990 n.s.
WT-Session 10 vs. PVKO-Session 1	−8.321	16.178	40.678	0.305 n.s.
WT-Session 10 vs. PVKO-Session 10	21.653	46.153	70.652	< 0.001 ***
PVKO-Session 1 vs. PVKO-Session 10	5.475	29.974	54.474	0.011 *
**Band 4-12 Hz: Many Groups**	**Statistical Tests and Reports**			***P*-value**
WT-Session 1 vs. WT-Session 10 vs. PVKO-Session 1 vs. PVKO-Session 10	One-Way ANOVA *F*-test: $F_{(3,44)}$ = 21.67<br>Tukey-Kramer test for the multiple comparisons			< 0.001 ***
**Band 4-12 Hz: Pairwise comparison**	**Confidence Interval (95%)**			
WT-Session 1 vs. WT-Session 10	−91.086	−65.796	−40.506	< 0.001 ***
WT-Session 1 vs. PVKO-Session 1	−61.168	−35.878	−10.587	0.002 **
WT-Session 1 vs. PVKO-Session 10	−27.635	−2.345	22.946	0.995 n.s.
WT-Session 10 vs. PVKO-Session 1	4.628	29.918	55.209	0.015 *
WT-Session 10 vs. PVKO-Session 10	38.161	63.451	88.742	< 0.001 ***
PVKO-Session 1 vs. PVKO-Session 10	8.243	33.533	58.823	0.005 **
**Band 12-30 Hz: Many Groups**	**Statistical Tests and Reports**			***P*-value**
WT-Session 1 vs. WT-Session 10 vs. PVKO-Session 1 vs. PVKO-Session 10	One-Way ANOVA *F*-test: $F_{(3,44)}$ = 14.97<br>Tukey-Kramer test for the multiple comparisons			< 0.001 ***
**Band 12-30 Hz: Pairwise comparison**	**Confidence Interval (95%)**			
WT-Session 1 vs. WT-Session 10	−30.106	−20.850	−11.593	< 0.001 ***
WT-Session 1 vs. PVKO-Session 1	−22.534	−13.278	−4.022	0.002 **
WT-Session 1 vs. PVKO-Session 10	−12.713	−3.456	5.800	0.752 n.s.
WT-Session 10 vs. PVKO-Session 1	−1.684	7.572	16.828	0.144 n.s.
WT-Session 10 vs. PVKO-Session 10	8.137	17.393	26.650	< 0.001 ***
PVKO-Session 1 vs. PVKO-Session 10	0.565	9.821	19.078	0.034 *
**Band 30-50 Hz: Many Groups)**	**Statistical Tests and Reports**			***P*-value**
WT-Session 1 vs. WT-Session 10 vs. PVKO-Session 1 vs. PVKO-Session 10	One-Way ANOVA *F*-test: $F_{(3,44)}$ = 26.66<br>Tukey-Kramer test for the multiple comparisons			< 0.001 ***
**Band 30-50 Hz: Pairwise comparison**	**Confidence Interval (95%)**			
WT-Session 1 vs. WT-Session 10	−14.764	−9.006	−3.248	< 0.001 ***
WT-Session 1 vs. PVKO-Session 1	−15.856	−10.098	−4.340	< 0.001 ***
WT-Session 1 vs. PVKO-Session 10	0.760	6.518	12.276	0.021 *
WT-Session 10 vs. PVKO-Session 1	−6.850	−1.092	4.666	0.957 n.s.
WT-Session 10 vs. PVKO-Session 10	9.766	15.524	21.282	< 0.001 ***
PVKO-Session 1 vs. PVKO-Session 10	10.858	16.616	22.374	< 0.001 ***
**Band 50-150 Hz: Many Groups**	**Statistical Tests and Reports**			***P*-value**
WT-Session 1 vs. WT-Session 10 vs. PVKO-Session 1 vs. PVKO-Session 10	One-Way ANOVA *F*-test: $F_{(3,44)}$ = 97.76<br>Tukey-Kramer test for the multiple comparisons			< 0.001 ***
**Band 50-150 Hz: Pairwise comparison**	**Confidence Interval (95%)**			
WT-Session 1 vs. WT-Session 10	−4.753	−3.366	−1.980	< 0.001 ***
WT-Session 1 vs. PVKO-Session 1	−5.698	−4.311	−2.925	< 0.001 ***
WT-Session 1 vs. PVKO-Session 10	2.301	3.687	5.074	< 0.001 ***
WT-Session 10 vs. PVKO-Session 1	−2.332	−0.945	0.441	0.278 n.s.
WT-Session 10 vs. PVKO-Session 10	5.667	7.054	8.440	< 0.001 ***
PVKO-Session 1 vs. PVKO-Session 10	6.612	7.999	9.385	< 0.001 ***

Note that: n.s. indicates non-significant differences; *, $P < 0.05$; **, $P < 0.01$; ***, $P < 0.001$.

Table S6.1. Statistical reports corresponding to the multiple comparisons for the mean values of the LFP spectral powers measured **from the CA1** (see **Fig. 6B,C**) respect to the **baseline condition**.

**Fig. 6B: LFPs recorded from Beginning (two first min.) and End (two last min.) of Sessions 4 to 8.**				
**Band 1-4 Hz: Pairwise comparison**	**Confidence Interval (95%)**			***P*-value**
WT-Beginning vs. WT-Baseline	34.040	82.222	130.400	< 0.001 ***
WT-End vs. WT-Baseline	69.698	117.880	166.060	< 0.001 ***
PVKO-Beginning vs. PVKO-Baseline	12.789	60.972	109.150	0.005 **
PVKO-End vs. PVKO-Baseline	14.752	62.934	111.120	0.004 **
WT-Baseline vs. PVKO-Baseline	−31.719	16.463	64.646	0.915 n.s.
**Band 4-12 Hz: Pairwise comparison**	**Confidence Interval (95%)**			***P*-value**
WT-Beginning vs. WT-Baseline	113.790	163.340	212.900	< 0.001 ***
WT-End vs. WT-Baseline	168.160	217.710	267.270	< 0.001 ***
PVKO-Beginning vs. PVKO-Baseline	63.242	112.800	162.350	< 0.001 ***
PVKO-End vs. PVKO-Baseline	62.410	111.970	161.520	< 0.001 ***
WT-Baseline vs. PVKO-Baseline	−28.731	20.825	70.380	0.819 n.s.
**Band 12-30 Hz: Pairwise comparison**	**Confidence Interval (95%)**			***P*-value**
WT-Beginning vs. WT-Baseline	41.159	59.481	77.803	< 0.001 ***
WT-End vs. WT-Baseline	48.107	66.430	84.752	< 0.001 ***
PVKO-Beginning vs. PVKO-Baseline	8.250	26.572	44.895	< 0.001 ***
PVKO-End vs. PVKO-Baseline	8.428	26.751	45.073	< 0.001 ***
WT-Baseline vs. PVKO-Baseline	−22.243	−3.920	14.402	0.989 n.s.
**Band 30-50 Hz: Pairwise comparison**	**Confidence Interval (95%)**			***P*-value**
WT-Beginning vs. WT-Baseline	13.062	24.007	34.953	< 0.001 ***
WT-End vs. WT-Baseline	14.429	25.375	36.320	< 0.001 ***
PVKO-Beginning vs. PVKO-Baseline	1.474	12.419	23.364	0.017 *
PVKO-End vs. PVKO-Baseline	1.393	12.339	23.284	0.018 *
WT-Baseline vs. PVKO-Baseline	−8.841	2.104	13.049	0.993 n.s.
**Band 50-150 Hz: Pairwise comparison**	**Confidence Interval (95%)**			***P*-value**
WT-Beginning vs. WT-Baseline	2.773	6.266	9.758	< 0.001 ***
WT-End vs. WT-Baseline	2.774	6.266	9.759	< 0.001 ***
PVKO-Beginning vs. PVKO-Baseline	−0.443	3.049	6.541	0.121 n.s.
PVKO-End vs. PVKO-Baseline	−0.501	2.992	6.484	0.135 n.s.
WT-Baseline vs. PVKO-Baseline	−0.273	3.220	6.712	0.088 n.s.

**Fig. 6B: LFPs recorded from operant conditioning Session 1 vs. Session 10.**				
**Band 1-4 Hz: Pairwise comparison**	**Confidence Interval (95%)**			***P*-value**
WT-Session 1 vs. WT-Baseline	−14.593	23.201	60.996	0.472 n.s.
WT-Session 10 vs. WT-Baseline	80.086	117.880	155.680	< 0.001 ***
PVKO-Session 1 vs. PVKO-Baseline	24.623	62.417	100.210	< 0.001 ***
PVKO-Session 10 vs. PVKO-Baseline	25.140	62.934	100.730	< 0.001 ***
WT-Baseline vs. PVKO-Baseline	−21.331	16.463	54.257	0.994 n.s.
**Band 4-12 Hz: Pairwise comparison**	**Confidence Interval (95%)**			***P*-value**
WT-Session 1 vs. WT-Baseline	−29.597	11.956	53.509	0.958 n.s.
WT-Session 10 vs. WT-Baseline	176.160	217.710	259.260	< 0.001 ***
PVKO-Session 1 vs. PVKO-Baseline	−20.851	20.702	62.255	0.689 n.s.
PVKO-Session 10 vs. PVKO-Baseline	70.413	111.970	153.520	< 0.001 ***
WT-Baseline vs. PVKO-Baseline	−20.729	20.825	62.378	0.684 n.s.
**Band 12-30 Hz: Pairwise comparison**	**Confidence Interval (95%)**			***P*-value**
WT-Session 1 vs. WT-Baseline	1.933	19.757	37.582	0.021 *
WT-Session 10 vs. WT-Baseline	48.605	66.430	84.254	< 0.001 ***
PVKO-Session 1 vs. PVKO-Baseline	−14.124	3.701	21.525	0.990 n.s.
PVKO-Session 10 vs. PVKO-Baseline	8.926	26.751	44.575	< 0.001 ***
WT-Baseline vs. PVKO-Baseline	−21.745	−3.920	13.904	0.987 n.s.
**Band 30-50 Hz: Pairwise comparison**	**Confidence Interval (95%)**			***P*-value**
WT-Session 1 vs. WT-Baseline	11.061	21.793	32.525	< 0.001 ***
WT-Session 10 vs. WT-Baseline	14.642	25.375	36.107	< 0.001 ***
PVKO-Session 1 vs. PVKO-Baseline	4.107	14.839	25.571	0.002 **
PVKO-Session 10 vs. PVKO-Baseline	1.606	12.339	23.071	0.015 *
WT-Baseline vs. PVKO-Baseline	−8.628	2.104	12.836	0.992 n.s.
**Band 50-150 Hz: Pairwise comparison**	**Confidence Interval (95%)**			***P*-value**
WT-Session 1 vs. WT-Baseline	3.083	6.508	9.933	< 0.001 ***
WT-Session 10 vs. WT-Baseline	2.841	6.266	9.692	< 0.001 ***
PVKO-Session 1 vs. PVKO-Baseline	6.464	9.889	13.315	< 0.001 ***
PVKO-Session 10 vs. PVKO-Baseline	−0.434	2.992	6.417	0.121 n.s.
WT-Baseline vs. PVKO-Baseline	−0.206	3.220	6.645	0.077 n.s.

Note that: n.s. indicates non-significant differences; *, $P < 0.05$; **, $P < 0.01$; ***, $P < 0.001$.

Table S6.2. Statistical reports corresponding to the multiple comparisons for the mean values of the LFP spectral powers measured **from the CA1** (see **Fig. 6B** in the main text).

Fig. 6B: LFPs recorded from the Beginning (two first min.) and the End (two last min.) of Sessions 4 to 8.				
**Band 1-4 Hz: Many Groups**	**Statistical Tests and Reports**			***P*-value**
WT-Beginning vs. WT-End vs. PVKO-Beginning vs. PVKO-End	One-Way ANOVA *F*-test: $F_{(3,44)} = 6.14$ Tukey-Kramer test for the multiple comparisons			0.001 **
**Band 1-4 Hz: Pairwise comparison**	**Confidence Interval (95%)**			
WT-Beginning vs. WT-End	−88.566	−35.658	17.249	0.287 n.s.
WT-Beginning vs. PVKO-Beginning	−15.194	37.714	90.621	0.242 n.s.
WT-Beginning vs. PVKO-End	−17.156	35.751	88.659	0.285 n.s.
WT-End vs. PVKO-Beginning	20.464	73.372	126.280	0.003 **
WT-End vs. PVKO-End	18.502	71.410	124.320	0.004 **
PVKO-Beginning vs. PVKO-End	−54.870	−1.962	50.945	0.999 n.s.
**Band 4-12 Hz: Many Groups**	**Statistical Tests and Reports**			***P*-value**
WT-Beginning vs. WT-End vs. PVKO-Beginning vs. PVKO-End	One-Way ANOVA *F*-test: $F_{(3,44)} = 25.62$ Tukey-Kramer test for the multiple comparisons			< 0.001 ***
**Band 4-12 Hz: Pairwise comparison**	**Confidence Interval (95%)**			
WT-Beginning vs. WT-End	−100.100	−54.370	−8.640	0.014 *
WT-Beginning vs. PVKO-Beginning	25.637	71.367	117.100	< 0.001 ***
WT-Beginning vs. PVKO-End	26.470	72.200	117.930	< 0.001 ***
WT-End vs. PVKO-Beginning	80.008	125.740	171.470	< 0.001 ***
WT-End vs. PVKO-End	80.840	126.570	172.300	< 0.001 ***
PVKO-Beginning vs. PVKO-End	−44.897	0.833	46.563	0.999 n.s.
**Band 12-30 Hz: Many Groups**	**Statistical Tests and Reports**			***P*-value**
WT-Beginning vs. WT-End vs. PVKO-Beginning vs. PVKO-End	One-Way ANOVA *F*-test: $F_{(3,44)} = 42.90$ Tukey-Kramer test for the multiple comparisons			< 0.001 ***
**Band 12-30 Hz: Pairwise comparison**	**Confidence Interval (95%)**			
WT-Beginning vs. WT-End	−17.847	−6.948	3.951	0.335 n.s.
WT-Beginning vs. PVKO-Beginning	18.090	28.988	39.887	< 0.001 ***
WT-Beginning vs. PVKO-End	17.911	28.810	39.709	< 0.001 ***
WT-End vs. PVKO-Beginning	25.038	35.937	46.836	< 0.001 ***
WT-End vs. PVKO-End	24.860	35.758	46.657	< 0.001 ***
PVKO-Beginning vs. PVKO-End	−11.077	−0.178	10.721	0.999 n.s.
**Band 30-50 Hz: Many Groups)**	**Statistical Tests and Reports**			***P*-value**
WT-Beginning vs. WT-End vs. PVKO-Beginning vs. PVKO-End	One-Way ANOVA *F*-test: $F_{(3,44)} = 34.19$ Tukey-Kramer test for the multiple comparisons			< 0.001 ***
**Band 30-50 Hz: Pairwise comparison**	**Confidence Interval (95%)**			
WT-Beginning vs. WT-End	−6.754	−1.367	4.020	0.905 n.s.
WT-Beginning vs. PVKO-Beginning	8.305	13.692	19.079	< 0.001 ***
WT-Beginning vs. PVKO-End	8.386	13.773	19.160	< 0.001 ***
WT-End vs. PVKO-Beginning	9.673	15.059	20.446	< 0.001 ***
WT-End vs. PVKO-End	9.753	15.140	20.527	< 0.001 ***
PVKO-Beginning vs. PVKO-End	−5.306	0.081	5.468	0.999 n.s.
**Band 50-150 Hz: Many Groups**	**Statistical Tests and Reports**			***P*-value**
WT-Beginning vs. WT-End vs. PVKO-Beginning vs. PVKO-End	One-Way ANOVA *F*-test: $F_{(3,44)} = 50.0$ Tukey-Kramer test for the multiple comparisons			< 0.001 ***
**Band 50-150 Hz: Pairwise comparison**	**Confidence Interval (95%)**			
WT-Beginning vs. WT-End	−1.994	−0.001	1.993	0.999 n.s.
WT-Beginning vs. PVKO-Beginning	4.443	6.436	8.429	< 0.001 ***
WT-Beginning vs. PVKO-End	4.500	6.493	8.487	< 0.001 ***
WT-End vs. PVKO-Beginning	4.444	6.437	8.430	< 0.001 ***
WT-End vs. PVKO-End	4.501	6.494	8.487	< 0.001 ***
PVKO-Beginning vs. PVKO-End	−1.936	0.057	2.051	0.999 n.s.

Note that: n.s. indicates non-significant differences; *, $P < 0.05$; **, $P < 0.01$; ***, $P < 0.001$.

Table S6.3. Statistical reports corresponding to the multiple comparisons for the mean values of the LFP spectral powers measured **from the CA1** (see **Fig. 6C** in the main text) .

**Fig. 6B: LFPs recorded from operant conditioning Session 1 vs. Session 10**				
**Band 1-4 Hz: Many Groups**	**Statistical Tests and Reports**			***P*-value**
WT-Session 1 vs. WT-Session 10 vs. PVKO-Session 1 vs. PVKO-Session 10	One-Way ANOVA *F*-test: $F_{(3,44)}$ = 14.26 Tukey-Kramer test for the multiple comparisons			< 0.001 ***
**Band 1-4 Hz: Pairwise comparison**	**Confidence Interval (95%)**			
WT-Session 1 vs. WT-Session 10	−135.800	−94.679	−53.563	< 0.001 ***
WT-Session 1 vs. PVKO-Session 1	−63.869	−22.752	18.364	0.459 ; n.s.
WT-Session 1 vs. PVKO-Session 10	−64.386	−23.270	17.847	0.440 ; n.s.
WT-Session 10 vs. PVKO-Session 1	30.811	71.927	113.040	< 0.001 ***
WT-Session 10 vs. PVKO-Session 10	30.293	71.410	112.530	< 0.001 ***
PVKO-Session 1 vs. PVKO-Session 10	−41.634	−0.517	40.599	0.999 ; n.s.
**Band 4-12 Hz: Many Groups**	**Statistical Tests and Reports**			***P*-value**
WT-Session 1 vs. WT-Session 10 vs. PVKO-Session 1 vs. PVKO-Session 10	One-Way ANOVA *F*-test: $F_{(3,44)}$ = 120.75 Tukey-Kramer test for the multiple comparisons			< 0.001 ***
**Band 4-12 Hz: Pairwise comparison**	**Confidence Interval (95%)**			
WT-Session 1 vs. WT-Session 10	−240.200	−205.760	−171.310	< 0.001 ***
WT-Session 1 vs. PVKO-Session 1	−22.362	12.079	46.520	0.786 n.s.
WT-Session 1 vs. PVKO-Session 10	−113.630	−79.185	−44.744	< 0.001 ***
WT-Session 10 vs. PVKO-Session 1	183.390	217.830	252.280	< 0.001 ***
WT-Session 10 vs. PVKO-Session 10	92.129	126.570	161.010	< 0.001 ***
PVKO-Session 1 vs. PVKO-Session 10	−125.700	−91.264	−56.823	< 0.001 ***
**Band 12-30 Hz: Many Groups**	**Statistical Tests and Reports**			***P*-value**
WT-Session 1 vs. WT-Session 10 vs. PVKO-Session 1 vs. PVKO-Session 10	One-Way ANOVA *F*-test: $F_{(3,44)}$ = 95.02 Tukey-Kramer test for the multiple comparisons			< 0.001 ***
**Band 12-30 Hz: Pairwise comparison**	**Confidence Interval (95%)**			
WT-Session 1 vs. WT-Session 10	−56.493	−46.673	−36.852	< 0.001 ***
WT-Session 1 vs. PVKO-Session 1	2.315	12.136	21.956	0.010 *
WT-Session 1 vs. PVKO-Session 10	−20.735	−10.914	−1.093	0.024 *
WT-Session 10 vs. PVKO-Session 1	48.988	58.808	68.629	< 0.001 ***
WT-Session 10 vs. PVKO-Session 10	25.938	35.758	45.579	< 0.001 ***
PVKO-Session 1 vs. PVKO-Session 10	−32.871	−23.050	−13.229	< 0.001 ***
**Band 30-50 Hz: Many Groups)**	**Statistical Tests and Reports**			***P*-value**
WT-Session 1 vs. WT-Session 10 vs. PVKO-Session 1 vs. PVKO-Session 10	One-Way ANOVA *F*-test: $F_{(3,44)}$ = 31.82 Tukey-Kramer test for the multiple comparisons			< 0.001 ***
**Band 30-50 Hz: Pairwise comparison**	**Confidence Interval (95%)**			
WT-Session 1 vs. WT-Session 10	−8.407	−3.582	1.244	0.210 n.s.
WT-Session 1 vs. PVKO-Session 1	4.232	9.058	13.883	< 0.001 ***
WT-Session 1 vs. PVKO-Session 10	6.733	11.558	16.384	< 0.001 ***
WT-Session 10 vs. PVKO-Session 1	7.814	12.639	17.465	< 0.001 ***
WT-Session 10 vs. PVKO-Session 10	10.315	15.140	19.966	< 0.001 ***
PVKO-Session 1 vs. PVKO-Session 10	−2.325	2.501	7.326	0.516 n.s.
**Band 50-150 Hz: Many Groups**	**Statistical Tests and Reports**			***P*-value**
WT-Session 1 vs. WT-Session 10 vs. PVKO-Session 1 vs. PVKO-Session 10	One-Way ANOVA *F*-test: $F_{(3,44)}$ = 47.34 Tukey-Kramer test for the multiple comparisons			< 0.001 ***
**Band 50-150 Hz: Pairwise comparison**	**Confidence Interval (95%)**			
WT-Session 1 vs. WT-Session 10	−1.602	0.242	2.085	0.985 n.s.
WT-Session 1 vs. PVKO-Session 1	−2.005	−0.162	1.681	0.995 n .s.
WT-Session 1 vs. PVKO-Session 10	4.893	6.736	8.579	< 0.001 ***
WT-Session 10 vs. PVKO-Session 1	−2.247	−0.404	1.440	0.936 n.s.
WT-Session 10 vs. PVKO-Session 10	4.651	6.494	8.337	< 0.001 ***
PVKO-Session 1 vs. PVKO-Session 10	5.054	6.898	8.741	< 0.001 ***

Note that: n.s. indicates non-significant differences; *, $P < 0.05$; **, $P < 0.01$; ***, $P < 0.001$.
